# Surgeon Recommendation and Outcomes of Decompression With vs Without Fusion in Patients With Degenerative Spondylolisthesis

**DOI:** 10.1001/jamanetworkopen.2024.53466

**Published:** 2025-01-07

**Authors:** Andreas Seip, Christian Hellum, Morten Wang Fagerland, Tore Solberg, Jens Ivar Brox, Kjersti Storheim, Erland Hermansen, Clemens Weber, Helena Brisby, Hasan Banitalebi, Håvard Furunes, Kari Indrekvam, Inger Ljøstad, Ivar Magne Austevoll

**Affiliations:** 1 Kysthospitalet in Hagevik, Orthopedic Department, Haukeland University Hospital, Bergen, Norway; 2 Division of Orthopedic Surgery, Oslo University Hospital Ullevål, Oslo, Norway; 3 Oslo Centre for Biostatistics and Epidemiology, Research Support Services, Oslo, Norway; 4 Institute of Clinical Medicine, The Arctic University of Norway UiT, Tromsø, Norway; 5 The Norwegian Registry for Spine Surgery (NORspine), University Hospital of North Norway, Tromsø, Norway; 6 Department of Physical Medicine and Rehabilitation, Oslo University Hospital, Oslo, Norway; 7 Medical Faculty, University of Oslo, Oslo, Norway; 8 Department of Rehabilitation Science and Health Technology, Faculty of Health Sciences, Oslo Metropolitan University, Oslo, Norway; 9 Research and Communication Unit for Musculoskeletal Health, Division of Clinical Neuroscience, Oslo University Hospital, Oslo, Norway; 10 Department of Research and Innovation, Møre and Romsdal Hospital Trust, Ålesund, Norway; 11 Institute of Health Sciences, Norwegian University of Technology and Science, Ålesund, Norway; 12 Department of Neurosurgery, Stavanger University Hospital, Stavanger, Norway; 13 Department of Quality and Health Technology, Faculty of Health Sciences, University of Stavanger, Stavanger, Norway; 14 Department of Orthopedics, Sahlgrenska University Hospital, Gothenborg, Sweden; 15 Institute of Clinical Sciences, Sahlgrenska Academy, University of Gothenburg, Sweden; 16 Department of Diagnostic Imaging, Akershus University Hospital, Lorenskog, Norway; 17 Institute of Clinical Medicine, University of Oslo, Oslo, Norway; 18 Department of Orthopedic Surgery, Innlandet Hospital Trust, Gjøvik, Norway; 19 Department of Clinical Medicine, University of Bergen, Bergen, Norway; 20 Norwegian Back and Spine Patients Association, Oslo, Norway

## Abstract

**Importance:**

The ability of surgeons to choose the right patient for fusion in addition to decompression when operating for degenerative spondylolisthesis with symptomatic spinal stenosis is debated. The addition of fusion increases risk, morbidity, and costs but has been claimed to give better results for selected patients.

**Objective:**

To investigate whether following surgeons’ opinions regarding fusion was associated with clinical outcomes.

**Design, Setting, and Participants:**

This cohort study was conducted alongside the Norwegian Degenerative Spondylolisthesis and Spinal Stenosis randomized clinical trial, which showed noninferiority for decompression alone compared with decompression with fusion. From February 12, 2014, to December 18, 2017, trial surgeons from 16 Norwegian departments denoted their preferred treatment for 222 of 267 patients with symptomatic spinal stenosis and degenerative spondylolisthesis. For this analysis, the clinical outcomes of the patients who were and were not randomized to the recommended treatment were compared.

**Main Outcome and Measures:**

The primary outcome was a reduction of at least 30% from baseline to 2 years after surgery on the Oswestry Disability Index, ranging from 0 (no impairment) to 100 (maximum impairment). Secondary outcomes included the Zürich Claudication Questionnaire, leg and back pain scores, and the EuroQol 5-Dimension score.

**Results:**

Among 222 patients (155 [70%] female; mean [SD] age, 66.2 [7.7] years), decompression alone was recommended for 112 patients, of whom 59 received only decompression, and additional fusion for 110 patients, of whom 57 received fusion. At 2-year follow-up, 87 of 116 patients (75%) who received surgery in agreement with the surgeons’ recommendations and 77 of 106 (73%) who received surgery in disagreement with the surgeons’ recommendations reached the primary outcome (difference, 2.4 percentage points; 95% CI, −9.1 to 13.9 percentage points). All secondary outcomes were in the same direction as the primary outcome.

**Conclusions and Relevance:**

In this cohort study of 222 patients with degenerative spondylolisthesis who participated in a randomized clinical trial, surgeons’ recommendations were not associated with better outcomes than a random allocation when deciding between decompression alone and decompression with instrumented fusion. The results suggest that surgeons performing degenerative spondylolisthesis surgery could rely safely on evidence of operating with decompression alone, despite the conflict of expert opinion.

## Key Points

**Question:**

What is the association of surgeon recommendation for fusion in addition to decompression with outcomes among patients randomized to decompression with fusion vs decompression alone?

**Findings:**

In this cohort study conducted alongside a randomized clinical trial, including 222 patients with a 2-year follow-up, surgical treatment in agreement with surgeons’ prerandomization recommendations was not associated with improved clinical outcomes compared with treatment discordant with the surgeons’ recommendations.

**Meaning:**

These findings suggest that adding fusion to decompression based on individual patient evaluation might not improve clinical outcomes compared with following current evidence of decompression alone as the standard treatment.

## Teaser

This cohort study of patients with degenerative spondylolisthesis assesses whether following surgeon recommendations for decompression with fusion vs decompression alone was associated with superior clinical outcomes.

## Introduction

Degenerative spondylolisthesis is a forward slip of one vertebra relative to the vertebra below caused by age-related changes in the intervertebral disks, the facet joints, and the surrounding ligaments.^1^ A concomitant narrowed spinal canal (ie, spinal stenosis) often causes leg and back pain, especially in the upright position.^2^ Surgical decompression of the stenotic segment is recommended in patients with significant symptoms after nonsurgical treatment.^3^ It is still debated whether some patients may benefit from the addition of instrumented fusion (stabilization with screws, rods, and bone grafts to achieve a bony union between the slipped vertebra and the vertebra below) as part of standard treatment.^4^ Fusion may reduce the risk of further slippage and the recurrence of stenosis but can also lead to implant failure and constitutes an expansion of the procedure and increased costs.^2,5^

Evidence from other trials supports decompression alone as the recommended treatment.^6,7,8,9^ Randomized clinical trials are acknowledged as the ideal source of data for comparing treatment efficacy. Still, this study design may be limited by a lack of pragmatism.^10^ For instance, it discounts surgeons’ expertise regarding choice of treatment, particularly in heterogeneous patient populations, such as individuals with degenerative spondylolisthesis.^11^ Thus, one might hypothesize that patients will fare better if surgeons choose the most appropriate treatment for each individual based on clinical characteristics and radiologic variables.^4,10^ In this survey, alongside the Norwegian Degenerative Spondylolisthesis and Spinal Stenosis (NORDSTEN-DS) trial,^12^ we investigated whether following the spine surgeons’ recommendations for type of surgery resulted in superior clinical outcomes compared with contradictory treatment allocation.

## Methods

### Study Design and Overview

This cohort study is a secondary analysis of surgeons’ recommendations for optimal treatment performed alongside the NORDSTEN-DS trial, which is an investigator-initiated, multicenter, randomized, open-label, parallel group, noninferiority trial comparing patients with degenerative spondylolisthesis surgically treated with decompression alone or decompression with fusion.^6^ The Regional Committee for Medical and Health Research Ethics of Central Norway approved the trial. The trial protocol and the 2-year results of the primary analysis have been previously published.^6,13^ We defined the objective of this cohort study before the trial began and followed the statistical plan from the efficacy trial for the primary and secondary outcomes but did not include the plan in the published trial protocol. The statistical analysis plan for this study is available in Supplement 1. Oral and written information about the NORDSTEN-DS trial was provided, and patients who were willing to participate signed the trial consent form. This study followed the Strengthening the Reporting of Observational Studies in Epidemiology (STROBE) reporting guideline.^14^

### Participants

For the primary outcome of the trial, the sample size (232 patients) was based on an assumed noninferiority of decompression alone with a noninferiority margin of 15%.^6,13^ For this secondary analysis, sample size was determined by the completeness of the data for the survey question, constituting 222 patient-surgeon pairs. Patients were screened for eligibility by surgeons employed at 16 public orthopedic and neurosurgical clinics in Norway. These departments were responsible for 57% of the registered lumbar spine operations in Norway in 2014. Included patients had to be between 18 and 80 years of age with neurogenic claudication and/or radiating pain into the lower limbs for more than 3 months. All had spinal stenosis verified by magnetic resonance imaging (MRI) and a degenerative spondylolisthesis of at least 3 mm only at the index level assessed on standing plain radiographs. There was no upper limit for the size of the spondylolisthesis, and patients were included regardless of the degree of motion or angulation between vertebral bodies in flexion and extension radiographs. Patients were not included if they had a deformed nerve root in the intervertebral foramen on MRI (foraminal stenosis grade 3 according to Lee classification),^15^ were previously operated on at the index level, had a former fracture or fusion surgery in the thoracolumbar region, had an isthmic defect in the pars interarticularis, or had spondylolisthesis in more than 1 level. A full list of inclusion and exclusion criteria is provided in eTable 1 in Supplement 2.

Patients were referred by general practitioners for surgical evaluation and were screened for eligibility for inclusion in the NORDSTEN trials. Conservative and operative management were both discussed with the patients, and surgery was selected through shared decision-making between February 12, 2014, and December 18, 2017. In the checklist for inclusion, the surgeons answered the question, “Which surgical method do you consider best for this patient if not participating in this trial, decompression with or without an additional instrumented fusion?” The patients were unaware of the surgeons’ preferences.

### Randomization

Data were entered into the MedInsight database, stored at the Clinical Trial Unit at Oslo University Hospital, where patients were subsequently randomly assigned to undergo decompression surgery alone or decompression surgery with instrumented fusion in a 1:1 ratio. Randomization was stratified according to treatment center and fusion or nonfusion group but not according to the surgeons’ treatment preferences.

### Intervention Groups

All patients randomized to the surgeons’ recommended treatment type were categorized into the agreement group, whereas all patients with randomization not concordant with the surgeons’ preferences were in the disagreement group. For the decompression alone group, a midline-preserving decompression was mandatory. The surgical technique for the fusion group was decompression technique at the surgeon’s discretion, instrumented fusion with pedicle screws and rods, mandatory posterolateral bone grafting, and optional interbody fusion. Participating surgeons were consultants with a singular or major focus on spinal surgery and were experienced in the trial’s surgical procedures. Before the start of the trial, investigators from the scientific board visited the departments and had regular meetings to ensure a common understanding of the techniques. Further details on the surgical data are given in eTable 2 in Supplement 2.

### Outcomes

We used the same patient-reported outcomes as the primary analysis,^6^ all translated and validated for the Norwegian population.^16,17,18^ The outcomes were predefined in a statistical analysis plan, published before database lock and unblinding. The primary outcome was a reduction of at least 30% in the Oswestry Disability Index (ODI) score, version 2.0, from baseline to 2-year follow-up.^19,20^ The ODI comprises 10 items, with a total score ranging from 0 (no impairment) to 100 (maximum impairment). Secondary outcomes were mean change from baseline to 2 years of follow-up for ODI scores, the Zürich Claudication Questionnaire responses,^21^ the numeric rating scale scores for leg pain and back pain,^22^ and the EuroQol 5-Dimension^23^ score. The Zürich Claudication Questionnaire is a disease-specific questionnaire and assesses symptom severity (range of 1 to 5, indicating best to worse), functional impairment (range of 1 to 4, indicating best to worse), and satisfaction with treatment (range of 1 to 4, indicating highest to lowest satisfaction). The numeric rating scale for leg pain and back pain assesses pain during the past week on an 11-point Likert scale (range of 0 to 10, indicating lowest to highest pain).^22^ The EuroQol 5-Dimension 3-Level questionnaire^22^ assesses health-related quality of life on 5 dimensions (mobility, self-care, usual activities, pain and discomfort, and anxiety and depression). As post hoc outcomes, to present the durability of treatments according to the surgeons’ preferences, we assessed the percentage of patients who reported their condition as “somewhat worse,” “much worse,” or “worse than ever” on the Global Perceived Effect scale (a 7-point Likert scale) and the incidence of subsequent surgery at the index or adjacent lumbar level.

### Statistical Analysis

All analyses were conducted between January 3, 2023, and February 24, 2023, in a modified intention-to-treat set (ie, patients who were operated on in accordance with the randomization and had available data after randomization). The assumption of normally distributed data was confirmed by visual inspection of histograms and descriptive statistics.

The primary outcome (ODI improvement ≥30%) and the sensitivity analysis were the same as in the analyses of treatment efficacy.^6^ The primary research hypothesis was that the clinical results during the 2-year follow-up were similar between the agreement and disagreement groups. Missing data on the primary outcome were imputed with the use of multiple imputations (ie, 50 imputations by chained equations). Additional details regarding imputation are provided in section 5.2.1 in the eAppendix in Supplement 2. For the primary outcome, we performed 3 sensitivity analyses: 1 in the per-protocol set, which consisted of all patients who had not undergone a secondary operation and had available data on the primary outcome (ODI at baseline and 2-year follow-up); 1 with complete cases (patients with available data on the primary outcome); and 1 in which missing data at 2-year follow-up were replaced with data at 1 year, if available.

In 2 robustness analyses, 1 for all patients for whom surgeons preferred decompression alone and 1 for patients for whom surgeons preferred decompression with fusion, we compared the mean ODI scores from baseline to 2-year follow-up for those operated on in agreement and those not operated on in agreement with the surgeon’s choices. To identify whether baseline variables were associated with the surgeon’s preferred treatment, we performed a multivariable logistic regression analysis. On the basis of suggestions from current literature, we included the following variables in the model: age, sex, smoking habits, body mass index, predominant back pain, the degree of spondylolisthesis, the presence of radiologic dynamic instability (ie, increase in slippage or increased segmental angulation on dynamic radiographs), disk height, facet joint orientation, and the presence of facet joint fluid.^24,25^

We analyzed binary outcomes using Newcombe hybrid score CIs.^26^ For the repeated-measures continuous patient-reported outcome measure scores, we used linear mixed models to estimate mean values with 95% CIs at baseline, 3 months, and 2 years postoperatively; the change from baseline to 2 years; and the between-group difference (with 95% CI) in change from baseline to 2 years. The models included fixed effects for treatment group, time, treatment group × time interaction, and trial center. Time was modeled as piecewise linear, with a knot at 3 months. A random intercept at the patient level was used. Because we estimated the effectiveness of being operated on in agreement or not with the surgeon’s recommendation, which was assumed to depend on an overall evaluation by the surgeon of the individual characteristics of each patient (eg, age, sex, leg and back pain intensity, and radiologic parameters), the statistical analyses were not adjusted for baseline characteristics. There was no method for adjustment of CIs for multiple comparisons of outcomes. These results are presented as point estimates with unadjusted 95% CIs. The widths of CIs were evaluated to indicate what size of associations comply with the data. The analyses were performed with the use of Stata/SE software, version 17 (StataCorp LLC).

## Results

**Figure 1.**
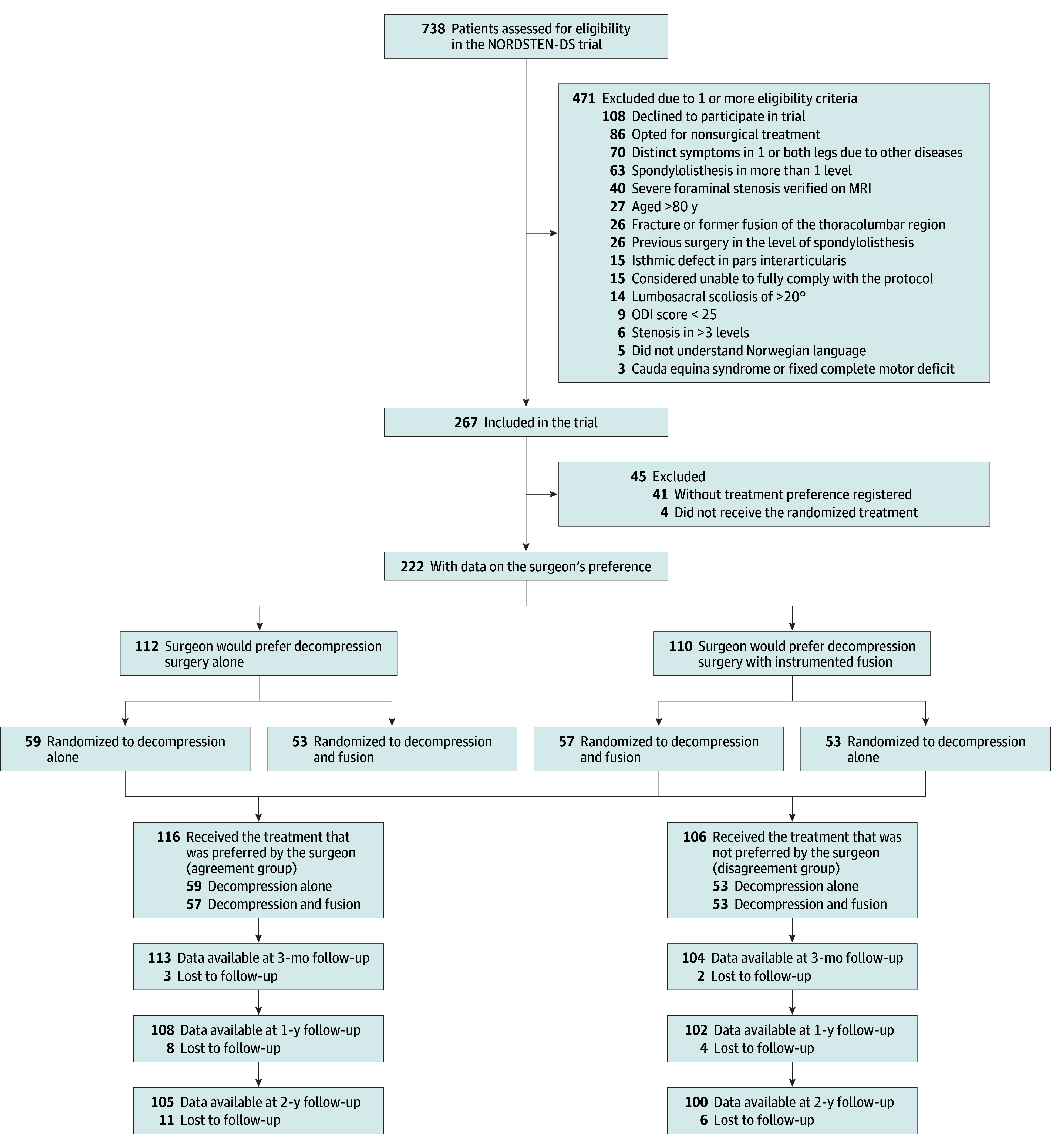
Allocation to Agreement and Disagreement Group Following the Trial Randomization MRI indicates magnetic resonance imaging; NORDSTEN-DS, Norwegian Degenerative Spondylolisthesis and Spinal Stenosis; ODI, Oswestry Disability Index.

A total of 267 patients were enrolled in the NORDSTEN-DS trial, of whom 226 had data on the surgeons’ preferences. Four patients were not operated on according to the randomization, leaving 222 patients in the modified intention-to-treat set (155 [70%] female and 67 male [30%]; mean [SD] age, 66.2 [7.7] years). The question on preferred treatment was not answered for 41 of 267 patients (15%). The surgeons preferred decompression alone for 112 patients and fusion for 110 patients (Figure 1; eTable 4 in Supplement 2).

Of the 112 patients for whom the surgeons recommended decompression alone, 59 (53%) were randomly assigned to this treatment. Of the 110 patients for whom the surgeons suggested fusion surgery, 57 (52%) were randomly assigned to fusion. Thus, there were 116 patients in the agreement group (57 fusion [49%]; 80 [69%] female; mean [SD] age, 66.1 [7.6] years) and 106 in the disagreement group (53 fusion [50%]; 77 [73%] female; mean [SD] age, 66.3 [7.4] years). Table 1 shows the epidemiologic, radiologic, and clinical parameters of the groups.

**Table 1.** Characteristics of the Patients at Baseline (Modified Intention-to-Treat Set)

Characteristic	No. (%) of patients^a^			
	Preferred procedure		Randomized group	
	Decompression alone (n = 112)	Decompression and fusion (n = 110)	Agreement group (n = 116)	Disagreement group (n = 106)
Age, mean (SD), y	66.3 (7.8)	66.1 (7.5)	66.1 (7.6)	66.3 (7.3)
Sex				
Female	73/112 (65)	82/110 (75)	80/116 (69)	77/106 (73)
Male	39/112 (35)	28/110 (25)	36/116 (31)	29/106 (27)
Smoker	25/111 (23)	14/107 (13)	21/113 (19)	18/105 (17)
BMI, mean (SD)	27.6 (4.7)	28.2 (4.2)	27.5 (4.7)	28.3 (4.3)
ASA score^b^				
ASA 1	11/108 (10)	14/109 (13)	16/113 (14)	9/104 (9)
ASA 2	76/108 (70)	82/109(75)	82/113 (73)	76/104 (73)
ASA 3	21/108 (19)	13/109 (12)	15/113 (13)	19/104 (18)
Degree of spondylolisthesis, mean (SD), mm^c^	6.5 (2.7)	8.2 (3.1)	7.2. (3.0)	7.5 (3.0)
Spondylolisthesis ≥20%^d^	21/98 (21)	48/101 (48)	35/104 (34)	34/95 (36)
Segmental instability^e^	15/103 (15)	27/103 (26)	20/105 (19)	22/101 (22)
Dynamic forward translation, mean (SD), mm	1.2 (1.6)	1.3 (2.0)	1.4 (1.6)	1.1 (1.7)
Increased local angulation in dynamic standing radiographs, mean (SD), °	2.8 (3.6)	3.8 (4.0)	3.1 (3.9)	3.5 (3.7)
Facet joint fluid, mean (SD), mm^f^	1.2 (0.9)	1.1 (1.1)	1.2 (1.0)	1.2 (1.0)
Orientation of the facet joint, mean (SD), °^g^	57 (8.5)	57 (8.6)	57 (8.8)	57 (8.1)
Disk height in the level of olisthesis, mean (SD), mm^h^	8.2 (3.4)	7.8 (1.9)	7.8 (2.1)	8.2 (3.3)
Lumbar lordosis^i^	53 (12)	55 (11)	53.8 (11.6)	55.0 (10.6)
ODI^j^	39.0 (13.3)	41.3 (13.5)	38.5 (12.4)	42.0 (14.3)
ZCQ symptom severity^k^	3.4 (0.6)	3.4 (0.5)	3.3 (0.5)	3.5 (0.6)
ZCQ physical function^k^	2.5 (0.5)	2.6 (0.5)	2.5 (0.5)	2.6 (0.5)
NRS leg pain^l^	6.6 (1.9)	6.9 (2.0)	6.5 (2.0)	7.0 (1.9)
NRS back pain^l^	6.5 (2.0)	7.0 (1.9)	6.4 (1.9)	7.1 (1.9)
EQ-5D-3L^m^	0.42 (0.30)	0.39 (0.32)	0.41 (0.31)	0.40 (0.30)

Abbreviations: ASA, American Society of Anesthesiologists; BMI, body mass index (calculated as weight in kilograms divided by height in meters squared); EQ-5D-3L, EuroQOL 5-Dimension 3-Level; NRS, numeric rating scale; ODI, Oswestry Disability Index; ZCQ, Zürich Claudication Questionnaire.

^a^ Unless otherwise indicated.

^b^ Score of 1 indicates no disease; 2, mild systemic disease; and 3, severe systemic disease that is not life-threatening.

^c^ The degree of spondylolisthesis refers to the forward translation of the upper vertebra relative to the vertebra below assessed on standing standard radiographs in millimeters.

^d^ Spondylolisthesis of 20% or more refers to a degree of the spondylolisthesis of more than 20% of the anteroposterior length of the upper end plate of the vertebra below assessed on standing standard radiographs.

^e^ Segmental instability refers to commonly used criteria for instability assessed on standing standard radiographs; an increase of slip 3 mm or more or segmental increase of angle between 2 vertebrae of 10° or more when bending forward from extended position.

^f^ Facet joint fluid refers to the gap between the upper end lower facets (mean of right and left) at the level of spondylolisthesis assessed by magnetic resonance imaging in the axial plane spaces.

^g^ Orientation of the facet joints refers to the angle of the facet joints (mean of right and left) relative to the coronary plane, assessed by magnetic resonance imaging in the axial plane spaces.

^h^ Disk height in the level of spondylolisthesis refers to the distance between midinferior and midsuperior disk borders assessed on a midsagittal magnetic resonance imaging plane.

^i^ Lumbar lordosis refers to the angle between upper end plate S1 and lower end plate L1 on standing standard radiographs.

^j^ The ODI comprises 10 items, with a total score ranging from 0 (no impairment) to 100 (maximum impairment).

^k^ The ZCQ assesses symptom severity (range of 1 to 5, indicating best to worse), functional impairment (range of 1 to 4, indicating best to worse), and satisfaction with treatment (range of 1 to 4, with lower scores indicating higher satisfaction).

^l^ The NRS assesses pain during the last week on an 11-point Likert scale with scores of 0 to 10, indicating lowest to highest).

^m^ The EQ-5D-3L assesses health-related quality of life that includes the domains of mobility, self-care, usual activity, pain or discomfort, and anxiety or depression, with scores of −0.59 to 1.0, indicating lowest to highest quality of life.

### Primary Outcome

**Figure 2.**
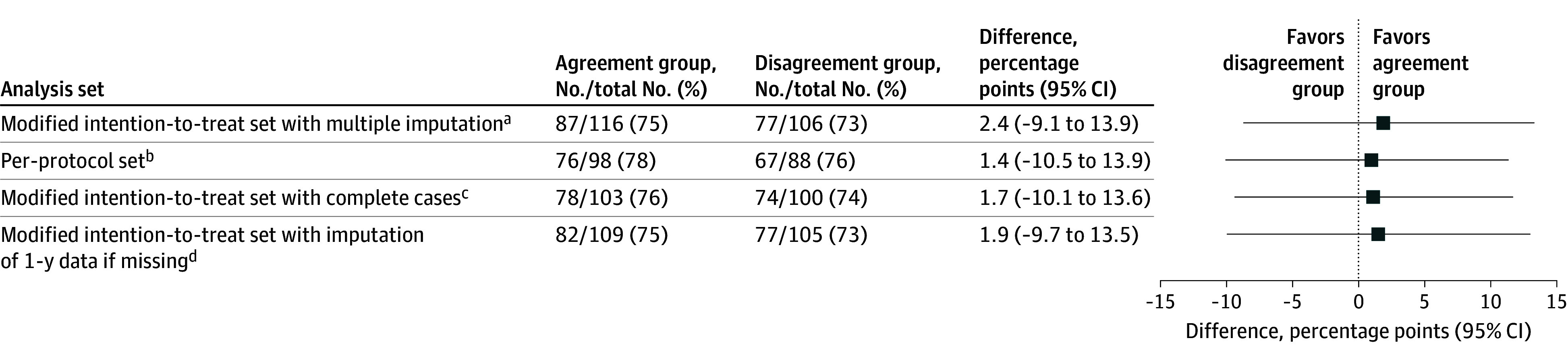
Results of the Primary Outcome The modified intention-to-treat set consisted of all the patients who received the trial treatment in accordance with the randomization assignment and had available data after randomization. The number of patients refers to the number who met the primary outcome (ie, had a reduction of ≥30% in the score on the Oswestry Disability Index [ODI] [range, 0-100, with higher scores indicating more impairment] from baseline to 2-year follow-up). The differences in percentage points and 95% CIs were calculated as agreement group minus decompression disagreement group. Error bars indicate 95% CIs. ^a^All the patients in the modified intention-to-treat set. Multiple imputation was performed if data on ODI were missing at baseline or at 2-year follow-up. ^b^Patients in the modified intention-to-treat set who did not undergo a subsequent lumbar operation during the follow-up period and who had available data on the primary outcome. ^c^Patients in the modified intention-to-treat set who had available data on the primary outcome. ^d^Patients in the modified intention-to-treat set for whom missing values on the ODI at 2-year follow-up were replaced by the values at 1 year, when available.

Of 222 patients, data on the primary outcome were missing for 2 patients (1%) at baseline and 17 (8%) at 2-year follow-up. In the modified intention-to-treat analysis with multiple imputation of missing data, 87 of 116 patients (75%) in the agreement group and 77 of 106 (73%) in the disagreement group (difference, 2.4 percentage points; 95% CI, −9.1 to 13.9 percentage points) had a reduction of at least 30% on the ODI from baseline to 2-year follow-up. The results of all sensitivity analyses were of similar size and in the same direction as the main analysis of the primary outcome, showing minor differences in estimates for group differences and CIs spanning the null effect with considerable margins on both sides (Figure 2).

### Secondary Outcomes

**Figure 3.**
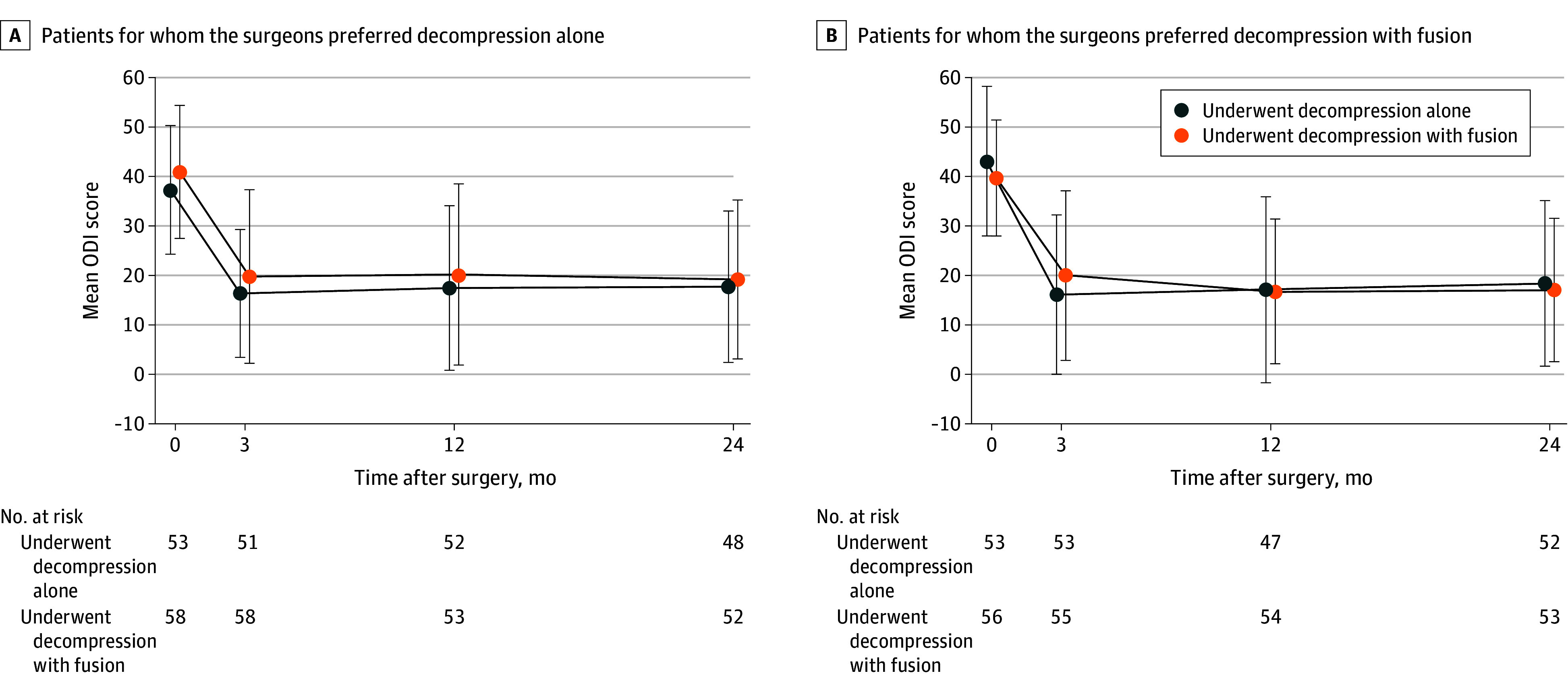
Oswestry Disability Index (ODI) Scores at Baseline and 3-Month, 1-Year, and 2-Year Follow-Up The modified intention-to-treat set with complete cases. Whiskers indicate SDs.

Table 2 gives the mean scores at baseline and 3- and 24-month follow-up and the mean changes from baseline to 24-month follow-up for the ODI. The mean changes in the ODI from baseline to 2-year follow-up were −21.4 (−24.1 to −18.7) points in the agreement group and −22.8 (−25.5 to −20.0) points in the disagreement group, for a mean difference of −1.36 (95% CI, −2.47 to 5.20). Similar minor differences in point estimates and CIs spanning the null between-group effect with considerable margins on both sides were found for other secondary outcomes. The mean change in the ODI was not associated with being operated on according to the surgeon’s recommendation, regardless of whether the surgeon recommended decompression alone or additional fusion (Figure 3).

**Table 2.** Results of the Linear Mixed-Model Analyses^a^

Outcome^b^	Mean (95% CI)				
	Baseline	3 mo	2 y	Change from baseline to 2 y	Difference in change, agreement vs disagreement
**ODI score** ^c^					
Agreement group	38.5 (35.7 to 41.3)	17.9 (15.3 to 20.6)	17.1 (14.3 to 19.8)	−21.4 (−24.1 to −18.7)	1.36 (−2.47 to 5.20)
Disagreement group	42.0 (39.2 to 44.9)	18.3 (15.5 to 21.0)	19.3 (16.4 to 22.1)	−22.8 (−25.5 to −20.0)	
**Score on ZCQ for symptom severity** ^d^					
Agreement group	3.31 (3.17 to 3.44)	2.16 (2.04 to 2.29)	2.33 (2.19 to 2.46)	−0.98 (−1.12 to −0.85)	0.10 (−0.09 to 0.30)
Disagreement group	3.50 (3.36 to 3.64)	2.25 (2.12 to 2.48)	2.42 (2.28 to 2.55)	−1.08 (−1.22 to −0.94)	
**Score on ZCQ for physical function** ^d^					
Agreement group	2.49 (2.38 to 2.60)	1.60 (1.49 to 1.70)	1.66 (1.55 to 1.77)	−0.83 (−0.95 to −0.71)	0.04 (−0.12 to 0.21)
Disagreement group	2.58 (2.47 to 2.70)	1.65 (1.54 to 1.76)	1.71 (1.60 to 1.82)	−0.87 (−0.99 to −0.75)	
**Score on ZCQ for patient satisfaction** ^d^					
Agreement group	NA	1.68 (1.55 to 1.81)	1.70 (1.56 to 1.83)	0.01 (−0.11 to 0.14)	−0.03 (−0.21 to 0.15)
Disagreement group	NA	1.77 (1.63 to 1.90)	1.80 (1.66 to 1.94)	0.04 (−0.08 to 0.17)	
**Score on NRS for leg pain** ^e^					
Agreement group	6.44 (5.99 to 6.90)	2.31 (1.88 to 2.74)	2.53 (2.08 to 2.98)	−3.91 (−4.44 to −3.38)	−0.14 (−0.90 to 0.62)
Disagreement group	7.06 (6.59 to 7.54)	2.86 (2.41 to 3.30)	3.29 (2.89 to 3.76)	−3.77 (−4.31 to −3.23)	
**Score on NRS for back pain** ^e^					
Agreement group	6.43 (5.99 to 6.87)	3.33 (2.92 to 3.75)	3.27 (2.84 to 3.71)	−3.15 (−3.66 to −2.65)	0.27 (−0.46 to 0.99)
Disagreement group	7.09 (6.63 to 7.55)	3.23 (2.80 to 3.66)	3.67 (3.22 to 4.12)	−3.42 (−3.94 to −2.90)	
**EQ-5D-3L score** ^f^					
Agreement group	0.41 (0.36 to 0.46)	0.70 (0.65 to 0.75)	0.73 (0.68 to 0.78)	0.32 (0.26 to 0.38)	0.02 (−0.06 to 0.11)
Disagreement group	0.40 (0.35 to 0.45)	0.71 (0.66 to 0.76)	0.69 (0.64 to 0.74)	0.29 (0.23 to 0.35)	

Abbreviations: EQ-5D-3L, EuroQol 5-Dimension 3-Level; NRS, numeric rating scale; ODI, Oswestry Disability Index; ZCQ, Zürich Claudication Questionnaire.

^a^ The modified intention-to-treat set consisted of all the participants who were operated on according to the randomization and had available data at baseline.

^b^ Shown are estimated mean values and mean changes for continuous repeated outcome measurements. Estimated values are based on linear mixed models. The CIs for differences between groups were not adjusted for multiple comparisons.

^c^ The ODI comprises 10 items, with a total score ranging from 0 (no impairment) to 100 (maximum impairment).

^d^ The ZCQ assesses symptom severity (range of 1 to 5, indicating best to worse), functional impairment (range of 1 to 4, indicating best to worse), and satisfaction with treatment (range of 1 to 4, with lower scores indicating higher satisfaction).

^e^ The NRS assesses pain during the past week on an 11-point Likert scale with scores of 0 to 10, indicating lowest to highest.

^f^ The EQ-5D-3L assesses health-related quality of life that includes the domains of mobility, self-care, usual activity, pain or discomfort, and anxiety or depression, with scores of −0.59 to 1.0, indicating lowest to highest quality of life.

### Durability and Surgeon Procedure Preference

According to the Global Perceived Effect scale, 7 of 105 patients (7%) in the agreement group and 7 of 100 (7%) in the disagreement group reported worsening of their condition at the 2-year follow-up. A subsequent operation at index level or adjacent lumbar level was performed in 7 of 114 patients (6%) in the agreement group (4 in the decompression preference group and 3 in the fusion preference group). The corresponding incidence in the disagreement group was 15 of 108 patients (14%; 8 in the decompression preference group and 7 in the fusion preference group) (eTable 5 in Supplement 2). The difference was 7.8% (95% CI, −0.2% to 16.1%).

The results of the multivariable logistic regression analysis (eTable 3 in Supplement 2) indicate that a higher degree of spondylolisthesis (odds ratio, 1.28 per mm; 95% CI, 1.13-1.45 per mm) on conventional standing radiographs and increased segmental angulation (OR, 1.13 per degree; 95% CI, 1.02-1.45 per degree) on dynamic standing radiographs were independently associated with the surgeon’s preference for fusion surgery.

## Discussion

In this secondary analysis alongside the NORDSTEN-DS trial, which involved 222 patients operated on for degenerative spondylolisthesis, the participating surgeons denoted which treatment they would prefer if the patients had not participated in the trial. The results showed that being operated on in agreement with the surgeons’ recommendation was not associated with improved clinical outcomes compared with treatment discordant with the surgeons’ recommendation. These findings remained robust in sensitivity and secondary outcome analyses as well as in separate analyses of patients recommended for decompression alone and for fusion surgery. Larger spondylolisthesis and increased segmental angulation in flexion were associated with the surgeons’ preference for additional fusion.

Previous studies have shown that spine surgeons consider a variety of factors in clinical decision-making (eg, sex, age, comorbidity, symptom patterns, and clinical and radiologic findings).^27,28,29,30^ It has been argued that randomized clinical trials may disguise subgroup effects and prohibit individualized treatment. Although a randomized clinical trial reveals no differences in efficacy between 2 treatment groups, the results could differ between subgroups determined by whether patients were treated according to participating surgeons’ recommendations.^10^ In the NORDSTEN-DS trial, there were no significant differences in 2-year clinical outcomes between decompression alone and decompression with fusion.^6^ However, the results of the agreement group could have differed from those of the disagreement group. For example, if patients with factors associated with the surgeons’ preference for fusion (eg, large spondylolisthesis and/or dynamic instability) had better outcomes with fusion surgery and those considered more appropriate for decompression alone (eg, patients with frailty or osteoporosis) benefited most from decompression alone, then treatment following the surgeon’s recommendation should give superior results compared with a random treatment allocation.

We have not identified any studies evaluating surgeons’ preferred choice of treatment in relation to clinical outcome after surgery for degenerative spondylolisthesis or any similar designs in other clinical fields. The inclusion of clinicians’ treatment preferences before randomization might add another dimension to a trial. Assessing how the treatments would perform if decision-making relied on the clinicians’ experience and expertise could enhance a trial’s external validity.

Similar to the current survey of the surgeons’ preferences, some surveys report that the degree of the slip and dynamic instability are considered reasons for adding fusion to decompression.^25,30^ Our analysis and previous studies show that Norwegian surgeons’ preferences were distributed equally between decompression alone and decompression with fusion.^31,32^ This result contrasts with reports from other countries.^32,33^

Despite some randomized clinical trials^6,7,8,9^ and meta-analyses^34,35,36^ concluding that fusion surgery seems to be superfluous for most patients operated on for degenerative spondylolisthesis, a shift in surgical practice has yet to occur.^37^ One reason may be that surgeons tend to maintain treatment traditions they are familiar with and rely on their own expertise to evaluate each patient. The lack of association between surgeons’ choices and clinical outcomes in the current study might indicate that commonly suggested factors are not helpful in determining whether a patient requires fusion. In the absence of known variables able to modify treatment effect,^38^ surgeons should rely on the evidence that adding fusion to decompression is unnecessary in most patients with degenerative spondylolisthesis.

Although evidence regarding treatment effect modifiers is sparse,^38^ we cannot rule out that some patients may benefit from additional fusion. Future high-quality studies are warranted to clarify the circumstances under which fusion surgery may be the most appropriate choice. In addition, it is important to recognize that the current study has not answered this question for patients who do not fulfill the inclusion criteria (eg, those with spondylolisthesis accompanied by severe foraminal stenosis or scoliosis, previous operations at the index level, spondylolisthesis in >1 level, and isthmic spondylolisthesis).

### Limitations

This study has several limitations. The trial size was not powered to explore differences in reoperation rates. The different point estimates of reoperations warrant further investigations on whether surgeon recommendations might influence the durability of surgery. The surgeons were not asked to elaborate on why they would prefer to add fusion or not. Although all contributing surgeons were trained in and familiar with both trial treatments, they might still have recommended the technique they were most confident in. Two of the included departments preferred decompression alone for all their patients, likely reflecting some clustering of the surgeons’ preferences.

There was no alternative response category on the surgeon inclusion form that indicated indifference to the procedure type. However, this reflects daily practice, where the surgeon must decide on a procedure. The question on preferred treatment was not answered for 41 of 267 patients (15%), introducing a risk of selection bias. Because all contributing surgeons were familiar with both midline-preserving decompression and fusion, the results might not be generalizable to surgeons unfamiliar with one of these techniques. Longer follow-up time is needed to reveal whether the surgeons’ preferences may still impact the clinical results.

## Conclusions

In this cohort study alongside the NORDSTEN-DS trial of patients with degenerative single-level spondylolisthesis, surgical treatment in agreement with the surgeons’ recommendations was not associated with improved patient-reported outcomes compared with treatment discordant with surgeons’ recommendations. The results suggest that surgeons performing degenerative spondylolisthesis surgery could rely safely on evidence of operating with decompression alone, despite the conflict of expert opinion.

## Notes

Supplement 1.
Statistical Analysis Plan

Supplement 2. **eAppendix.** The NORDSTEN Collaboration **eTable 1.** Study Eligibility Criteria **eTable 2.** Surgical and Radiological Data (Modified Intention to Treat Population for the Total NORDSTEN-DS Trial) **eTable 3.** Associations of Baseline Characteristics and the Surgeon’s Preferred Treatment Analyzed With Multivariable Logistic Regression Models (N = 179) **eTable 4.** Proportion of Preference at Participating Departments **eTable 5.** Worsening According to GPE Scale and Subsequent Surgery From Index Surgery to 2-Year Follow-Up
eReferences

Supplement 3.
Data Sharing Statement
