# Supplementary material for: Surgeon Recommendation and Outcomes of Decompression With vs Without Fusion in Patients With Degenerative Spondylolisthesis
Source: JAMA Netw Open. 2025 Jan 7;8(1):e2453466. doi: 10.1001/jamanetworkopen.2024.53466 (PMC11707628; doi:10.1001/jamanetworkopen.2024.53466)
Supplement: Supplement 1. — Statistical Analysis Plan [file jamanetwopen-e2453466-s001.pdf]

Statistical Analysis Plan

Surgeon Preference versus Random Allocation in Surgery for Degenerative Spondylolisthesis?

An analysis alongside the NORDSTEN-DS trial

SAP Version 1.0

Date: January 15, 2024

1. Administrative Information

This document is a supplement to the NORDSTEN-DS protocol; "Decompression alone versus decompression with instrumental fusion; The NORDSTEN Degenerative Spondylolisthesis Trial (NORDSTEN-DS); Study protocol for a randomized controlled trial".[1]

Trial Registration Number

The NORDSTEN-DS trial is registered in ClinicalTrials.gov, first received January 10, 2014 (Identifier: NCT02051374).

Contributors to current SAP

Andreas Seip, MD (1), E-mail: seip@helse-bergen.no
Ivar Magne Austevoll MD, PhD (1), E-mail: imau@helse-bergen.no
Morten Fagerland, Biostat, PhD (2) E-mail: morten.fagerland@medisin.uio.no

Sponsor/ Funding

The project has received funding from the Western Regional Health Authority, the Møre and Romsdal Hospital Trust, and the Sophies Minde foundation. The funder has no influence in study design, management and interpretation of data or in decision to submit data.

Signature Page

I hereby declare that I have reviewed and approved the Statistical Analysis Plan:

Principal investigator

Signature ____

Andreas Seip[1]

Date ____

Study chair

Signature ____

Ivar M Austevoll[1]

Date ____

Responsible Biostatician

Signature ____

Morten Wang Fagerland[2]

Date ____

[1] Kysthospitalet in Hagevik, Orthopedic Clinic, Haukeland University Hospital, Bergen, Norway

[2] Oslo Centre for Biostatistics and Epidemiology, Research Support Services, Oslo University Hospital

Table of contents

Abbreviations and definitions

DA	Decompression alone
DF	Decompression with instrumental fusion
DS	Degenerative spondylolisthesis
ITT	Intention to treat
LSS	Lumbar spinal stenosis
NNT	Number needed to treat
NRS	Numeric rating scale
MI	Multiple imputation
ODI	Oswestry disability index
PROM	Patient reported outcome
SAP	Statistical Analysis Plan
ZCQ	Zürich claudication questionnaire –score

Follow-up score = time-point value for the actual score
Change score = time point value – baseline value
Percentage change score = [(time-point value – baseline value) / baseline value] *100%

SAP revisions

The main content of the present SAP is in accordance with the SAP for the NORDSTEN-DS trial, the published study protocol, and the first registration in ClinicalTrials.gov January 10, 2014 (IdentiferNCT02051374). For the NORDSTEN-DS trial the following clarifications are declared in updates to clincaltrials.gov:

1. Update September, 2014

Four exclusion criteria was recorded in the original protocol but were not recorded in first registration in Clinical trials. However, these criteria were used for enrolling patients from the start of the study. The criteria added to the update were:
1. Previous surgery in the level of spondylolisthesis; 2. Lumbosacral scoliosis of more than 20 degrees verified on AP-view; 3. Distinct symptoms in one or both legs due to other diseases, e.g. polynevropathy, vascular claudication or osteoarthtritis; 4. Radicular pain due to a MRI-verified foraminal stenosis in the slipped level, with deformation of the nerve root because of a bony narrowing in the vertical direction.

2. Update January, 2016

From the start of inclusion (April 15, 2014) patients with ODI scores less than 25 were excluded. Due to experiences from participating surgeons that a considerable part of the patients were excluded due to ODI less than 25, even if their complaints from leg and back did justify an operation. To enhance the external validity of the study, the steering committee decided that from date 29th August 2015, the patients should not be excluded due to ODS- score lower than 25.

3. Update September, 2017

In accordance with the study “Follow-up score, change score or percentage change score for determining clinical important outcome following surgery?”[2] the criteria for a clinical important outcome assessed by the primary outcome (ODI) was recorded.
We also changed the plan for handling missing data in the primary outcome. Instead of using Multiple imputation we planned different ‘worse case – best case ’imputation scenarios for sensitivity analysis.

4. Update January 2020

After thorough discussions in the study group, and review of current literature, we decided to reintroduce the original planned method for handling missing data due to ‘lost to

follow-up'. Patients without measurement available for dichotomizing into responder/non-responder (ODI, ZCQ, NRS leg pain, and NRS back pain) will receive two-year follow-up scores estimated with use of Multiple Imputation.

Amendments.

No amendments have been made to the present protocol.

Statistical analyses of the present study

A superiority design will be used. The plan for the statistical analyses of the present study is in accordance with the plan for the NORDSTEN-DS efficacy trial. The primary outcome will be analysed where missing scores will be imputed by use of Multiple imputation (MI). Three sensitivity analysis will also be performed to evaluate the primary outcome. One in a Per-protocol Set; one using the modified ITT population with complete case analysis and one using the modified ITT population where missing values will be replaced with values at one year follow-up, if available.

2 Introduction

Background and rationale

Degenerative spondylolisthesis (DS) is defined as a forward slippage of one vertebra over another without a disruption in the vertebral arch[3]. In most occasions the patients present symptoms related to a concomitant spinal stenosis, typically back and leg pain in supine position[4,5].

The Norwegian degenerative spondylolisthesis and spinal stenosis (NORDSTEN)-DS trial, and other recent randomized trials (RCTs) have provided evidence for decompression alone as the recommended treatment.[6-8] Nevertheless, although RCTs are presumed to be the ideal source of data comparing treatment efficacy, limitations exist for this study design. One limitation might be the ability to assess the effectiveness of treatments on individuals.[9] Thus we might hypothesize that individual patients will fare better if the surgeons choose the most appropriate treatment for each patient, based on clinical characteristics, radiological variables or combinations of these.

The present document describes the planned statistical analysis plan for a secondary analysis, alongside the NORDSTEN-DS trial. We intend to investigate whether treatment following the spine surgeons 'preference for type of surgery resulted in superior clinical outcomes compared with treatment allocated opposite to the surgeon's opinion.

Objective

Main Objective

Before randomisation, the responsible surgeon denoted their preference for the most appropriate treatment (decompression alone or decompression with fusion) if the patient had not been participating in the trial.

The primary objective is to investigate whether following a surgeon's recommendation for treatment (decompression alone or decompression with fusion) gives superior results than if patients are being operated with the treatment the surgeon did not prefer.

Secondary objective

To possibly identify baseline variables associated with the surgeon's preferred treatment, we will perform a multiple logistic regression analysis. Based on suggestions from current literature[10] we will include the following variables in the model: age, gender, smoking habits, body mass index, predominant back pain, the degree of spondylolisthesis, the presence of radiological dynamic instability (i.e. increase in slippage or increased segmental angulation on dynamic radiographs), disc height, facet joint orientation, and the presence of facet joint fluid.

3. Study Methods

Trial overview

The NORDSTEN-DS trial is a 1:1 block- randomized, controlled, multicenter, one-country, non- inferiority trial, with two parallel groups.

This study is an analysis alongside the NORDSTEN- DS trial, a Norwegian multicentre study on patients with lumbar spinal stenosis. In NORDSTEN-DS decompression without fusion is being compared to decompression with instrumented fusion.

All patients willing to participate, fulfilled the trial consent form. Before randomisation, without involvement from the patients, the surgeons fulfilled the Checklist for inclusion, which included the following question: Which surgical method would be preferable for this patient if not in a trial, decompression with or without an additional fusion?

Sample size was determined by the original study, and by the completeness of the data for the survey question in the surgeon inclusion papers. The lack of sample size calculation could be considered a limitation to the present study

Trial treatments

Decompression alone: The midline (i.e., the spinous process and the interspinous ligaments) was preserved and one of the following techniques was used: 1) unilateral laminotomy; 2) bilateral laminotomy; 3) unilateral laminotomy and crossover decompression. Magnifying devices (microscope or loupes) will be used.

Decompression and instrumented fusion: A decompression with or without preservation of the midline structures, and additional posterior pedicle screw instrumental fusion with or without an intervertebral cage. Magnifying devices (microscope or loupes) was used.

Groups for comparison in this study

The Agreement group: All patients randomized in concordance with the treatment the surgeon preferred.

The Disagreement group: All patients with randomisation not concordant to the surgeons ’preference.

The SPIRIT checklist[11] has been used as a template for the original NORDSTEN-DS trial protocol.[1] The statistical analysis plan is prepared in accordance with guidelines for Statistical analysis plans in clinical trials.[12] The reporting of the trial will be based on an adapted Consolidated Standards of Reporting Trials (CONSORT) checklist for reporting randomized trials.[13]

The trial is monitored following the Helsinki Declaration, The International Conference on Harmonisation Guideline for Good Clinical Practice (ICH GCP).[14]

The protocol for NORDSTEN-DS has been approved by the Norwegian Committee for Medical and Health Research Ethics Midt (2013/366).

Randomization to treatment

The computer-generated randomisation was block- permuted and centre- stratified. The system used for patient allocation was Medinsight - a registry tool developed for researchers and health professionals by Institute for Cancer Genetics and Informatics at Oslo University Hospital, Norway. The patients were randomised in a 1:1 ratio to one of two arms (randomly selected block size 4 and 6). Randomisation was stratified to treatment centre and fusion/nonfusion, but not stratified to surgeons ’treatment preference.

After the patient signed the informed consent form, the randomization was performed within 6 weeks before treatment. The computer-generated randomization procedure was concealed and administered by a central coordinator.

Details of block size, allocation sequence generation, and randomization, was unavailable to those who enrolled patients or assign treatment.

Framework

Assessment of efficacy

The study is designed to establish the superiority in clinical outcomes for patients operated in agreement with the surgeons ’recommendation compared with patients randomized to the treatment the surgeon did not recommend. Test for superiority will be performed for the primary outcome (improvement in ODI ≥ 30%).

Secondary outcomes are mean scores at follow-up for ODI, NRS leg pain, NRS back pain, the Zürich Claudication Questionnaire, and the EuroQol Group 5-dimension questionnaire.

Blinding:

Patients were blinded for the surgeon's recommendation (i.e., they were unaware of information regarding surgeon preference denoted on the surgeon form). The treatment given (decompression alone or decompression with fusion) was not blinded for the patients. For analysis and testing of primary and secondary outcomes, the statistician will be blinded for treatment adherence.

Timing of outcome assessment

The study coordinators were responsible for the collection and administration of data at baseline and at 3-month follow-up. Data from 12-month 2-year, 5-year and 10-year follow-up is collected by the central coordinator at FORMI. All data are stored at the Faculty of Research support, University of Oslo. Time schedule for assessment of data in Nordsten-DS is presented in Table 2. For data assessed outside the time frame a "Note to file" were recorded in the patients 'Case Report Form (CRF).

Table 2. Time points at which the outcomes are measured for the NORDSTEN/DS trial

	Before operation	Hospital stay	3 months (±2 weeks)	12 months (±1 month)	2 years (±2 months)	5 years (±3 months)	10 years (±3 months)
Demographics	X[1]						
Lifestyles	X[1]						
PROMs	X[1]		X	X	X	X	X
X-rays	X[2]				X	X	X
MRI scan	X[2]						
CT scan					X		
Operation data		X					
Data from hospital stay		X					
Complications, reoperations		X	X	X	X	X	X

Abbrevations: MRI= Magnetic resonance imaging, CT= Computed tomography, AP= anterior- posterior, PROMs= Patient reported outcome measurements,
[1] Maximum 6 weeks prior operation date
[2] Maximum 6 months prior operation date

4. STATISTICAL PRINCIPLES

A significance level of 5% will be used throughout. For all analyses a 95% CI will be estimated and reported.

Adherence and protocol deviations

The trial was monitored following the Helsinki Declaration, The International Conference on Harmonisation Guideline for Good Clinical Practice (ICH GCP) [50]. An independent monitor, without influence on the scientific work, is responsible for the monitoring. Due to the non-regulated ICH GCP guideline for this trial (not including drug intervention) the risk and safety were safeguarded at the same level as data quality. All informed consent forms were checked, and all registrations of serious events were monitored. According to the monitoring plan selected variables were checked. All hospitals were visited regularly. Adapted versions of the ‘Investigator’s Site File (ISF) ’and the ‘Trial Master File (TMF) ’ were checked for essential documents during the trial. Queries and deviations were recorded and reported, and the coordinators at responsible hospitals had two months to send a written report with the required corrections to the monitor. All deviations from the protocol have subsequently been recorded at the ‘Note to file form’.

The patients have **major deviations from protocol if they**:

- have not received operative treatment in accordance with randomized allocation.
- have received operative treatment in accordance with randomized allocation and operated with a new lumbar operation during the follow-up period.
- have not provided informed consent.
- have withdrew the informed consent and claimed their data withdrawn from analyses.

According to the original SAP for the NORDSTEN efficacy trial the following analysis sets are defined:

Modified intention-to-treat (ITT) set: all randomised patients with primary operation according to the randomly assigned study treatment and with data on the primary outcome variable (ODI) at one or more time point.

Per-protocol set: All randomised patients without major deviations from protocol and with data on the primary outcome variable at baseline and two-year follow-up.

5 Trial population

Sixteen Norwegian orthopedic and neurosurgical hospital departments participate in the study.

Criteria for inclusion and exclusion are given in Table 2.

Table2

**Inclusion criteria:**<br>To be eligible for the study the participants must:	**Exclusion criteria:**<br>The participants will be excluded from the study if they:
Be over 18 years of age.<br>Understand Norwegian language, spoken and written.<br>Have a spondylolisthesis, with a slip >=3 mm, verified on standing plain x-rays in lateral view.<br>Have a spinal stenosis in the level of spondylolisthesis, shown on MRI, CT scan or myelogram.<br>Have clinical symptoms of spinal stenosis as neurogenic claudication or radiating pain into the lower limbs, not responding to at least 3 months of qualified conservative treatment.<br>Be able to give informed consent and to respond to the questionnaires.	Are not willing to give written consent.<br>Are participating in another clinical trial that may interfere with this trial.<br>Are ASA- grade > 3.<br>Are older than 80 years.<br>Are not able to fully comply with the protocol, including treatment, follow-up or study procedures (psychosocially, mentally and physically).<br>Have cauda equina syndrome (bowel or bladder dysfunction) or fixed complete motor deficit.<br>Have a slip >=3 mm in more than one level.<br>Have an isthmic defect in pars interarticularis.<br>Have a fracture or former fusion of the thoracolumbal region.<br>Have had previous surgery in the level of spondylolisthesis.<br>Have a lumbosacral scoliosis of more than 20 degrees verified on AP-view.<br>Have distinct symptoms in one or both legs due to other diseases, e.g. polynevropathy, vascular claudication or osteoarthtritis.<br>Have radicular pain due to a MRI-verified foraminal stenosis in the slipped level, with deformation of the nerve root because of a bony narrowing in the vertical direction.

MRI= Magnetic resonance imaging, CT= Computed tomography, AP= anterior- posterior, ASA = American Society of Anesthesiologists

All patients who the surgeons consider eligible at the participating hospitals were recorded. The surgeon recorded the results of the screening at the Screening Form. For patient fulfilled all eligible criteria and who have signed the consent form the surgeon completed form “Checklist for inclusion”, which included the following question: Which surgical method would be preferable for this patient if not in a trial, decompression with or without an additional fusion? Patients were not involved in the surgeons ’choice.

A CONSORT flow chart will be presented showing the number of patients included in the Agreement group and the Disagreement group.

List of baseline data to be summarized.

Demographics and lifestyles

- Age
- Gender
- Smoking habitus
- Body Mass Index
- American Society of Anesthesiologists (ASA) grade

Baseline PROMs

- Oswestry disability index score
- Zürich Claudication Questionnaire (ZCQ)
- Numeric Rating Scale for leg pain
- Numeric Rating Scale for back pain
- EuroQol Group 5-dimension

Radiological parameters

- Spondylolisthesis at standing x-rays in millimetres [15]
- Spondylolisthesis at standing x-rays Spondylolisthesis ≥ 20%
- Segmental instability[15]
- Amount of facet joint fluid [16]
- Orientation of the facet joint [17]
- Disc height in the level of spondylolisthesis [18]
- Lumbar lordosis [19]

Surgical data

- Level with spondylolisthesis
- Number of levels operated on
- Method used for decompression
- Method used for instrumented fusion

6. Analysis

Primary outcome

The primary outcome is an improvement of Oswestry Disability Index[20] (ODI) V.2.0[21] of more than 30% from baseline to 2 year follow-up. Based on former studies [22,23] and a study from The Norwegian Registry for Spine Surgery, an individual ODI improvement of 30% or more from baseline to follow-up has been chosen as the cut-off for being a responder [24]. We define patients with such improvement to be responders. The difference in the proportions of responders will be estimated with the Newcombe hybrid score CI [25].The null hypothesis (H0) is that the responder rate in the Agreement group is similar to the responder rate in the Disageement group (DG). H0 will be tested by forming a 95% confidence interval (CI) for the between-group difference in responder rate (responder rate Agreement group minus responder rate Disageement group and will be rejected if the lower bound of the CI is above zero. The alternative hypothesis is that Agreement group is superior to the Disagreement group.

Secondary outcomes

All results from analysis of secondary outcomes will be supplements to analyses of the primary outcome and discussed and interpreted accordingly.

Mean scores at the Oswestry Disability Index

For the continuous scores we will use linear mixed models to estimate mean scores at follow-ups and differences between the groups in mean change scores at 2-year follow-up. Because most change from baseline is expected to occur the first three months, the time development in the linear mixed models will be modelled as piecewise linear, with a knot at 3 months. The models will include fixed effects for allocation group (Agreement and Disagreement), time, treatment group x time interaction, and centre (stratification factor in the randomization). A random intercept will be used, and – if possible – a random effect for allocation group. Results of the analysis will be presented and interpreted.

Mean scores for Zürich Claudication Questionnaire (ZCQ)

ZCQ is a self-completed disorder-specific functional score consisting of three domains: symptom severity, physical function and patient satisfaction.

We will use linear mixed models (see above) to estimate mean scores at follow-ups and differences between the groups in mean change scores at 2-year follow-up.

Results of the analysis will be presented and interpreted.

Mean scores for Numeric Rating Scale (NRS) for back- and leg pain

NRS is a PROM that assesses self-reported pain the patients experienced in the last week from 0 (no pain) to 10 (the worst pain imaginable).

We will use linear mixed models (see above) to estimate mean scores at follow-ups and differences between the groups in mean change scores at 2-year follow-up.

Results of the analysis will be presented and interpreted.

Robustness analyses

Robustness analysis will be done for the subgroups of those recommended for decompression alone, and those recommended for decompression and addition of fusion. Outcome for this will be mean ODI at 3 and 6 months and 2 years. Results of the analysis will be presented and interpreted.

Analysis methods

All analyses described in this SAP will be applied and interpreted according to the description. Statistical methods are similar to the methods of the efficacy trial which have been defined in ClinicalTrials.gov, the published protocol and the SAP.

Descriptive statistics, including measures of centrality and variability, will be used to describe the baseline characteristics of the two treatment groups. The assumption of normal distribution will be checked by visual inspection of histograms.

Decision rules

Efficacy

The conclusion will be based on the modified ITT set, at two-year follow-up [26]. In the modified ITT population missing scores necessary for dichotomizing patients into responders/non-responders will be imputed by use of Multiple imputation (MI).

To not deviate from the SAP of the main NORDSTEN DS-trial, we will in addition perform three sensitivity analyses of the primary outcome. One in the Per-protocol Set population, one in the modified ITT without imputation (a complete cases analysis, and one in the modified ITT, where missing values will be replaced with values at one year follow-up, if available (FAS1-yearI).

Management of missing data

Although a strictly conducted study regarding routines for completing the follow-up questionnaires, some loss to follow-up was expected. Under the assumption of missingness at random (MAR), missing values necessary for estimating responder rates at two-year follow-up will be imputed by MI. The MAR assumption for patients not replying PROM-questionnaires is supported by a previous study from the Norwegian Registry for Spine Surgery [27]. Further, due to comprehensive set of available predictors for the imputation model, we consider the MI method robust regarding bias estimates. The imputation model, using linear regression, will include the following explanatory variables: Baseline patient characteristics (age; gender; education; first language; smoking; body mass index; former spinal surgery; duration of pain; use of analgesics), radiological parameters at baseline (degree of the slip; segmental instability; Schizas grade; orientation of facet joint; disc height), operation time, length of hospital stay, baseline and follow-up scores for ODI, NRS leg pain, NRS back pain, Eq-5D, ZCQ, GPE, duration of surgery, length of hospital stay, complications, and reoperation. The imputation will be carried out stratified by treatment (i.e., by treatment group separately) [28]. The multiply imputing will be performed before dichotomizing, as recommended [29]. It will be generated 50 data sets with complete two-year follow-up scores for ODI, ZCQ, NRS leg pain and NRS back pain. Before the responder analyses, which include the Newcombe hybrid score CI, the imputed scores will be estimated based on the 50 aggregated data sets.

Under the MAR assumption, the mean change and mean follow-up scores in continuous variables will be analysed by Linear mixed effect models, estimated with Full Information Maximum Likelihood.

7. References

1. Austevoll IM, Hermansen E, Fagerland M, et al. Decompression alone versus decompression with instrumental fusion the NORDSTEN degenerative spondylolisthesis trial (NORDSTEN-DS); study protocol for a randomized controlled trial. *BMC Musculoskelet Disord*. Jan 5 2019;20(1):7. doi:10.1186/s12891-018-2384-0
2. Austevoll IM, Gjestad R, Grotle M, et al. Follow-up score, change score or percentage change score for determining clinical important outcome following surgery? An observational study from the Norwegian registry for Spine surgery evaluating patient reported outcome measures in lumbar spinal stenosis and lumbar degenerative spondylolisthesis. *BMC Musculoskeletal Disorders*. 2019/01/18 2019;20(1):31. doi:10.1186/s12891-018-2386-y
3. Farfan HF. - The pathological anatomy of degenerative spondylolisthesis. A cadaver study. - *Spine (Phila Pa 1976)1980 Sep-Oct;5(5):412-8*. 3/12/2012 2012:Oct. Not in File.
4. Fitzgerald JA, Newman PH. Degenerative spondylolisthesis. *J Bone Joint Surg Br*. May 1976;58(2):184-92.
5. Watters WC, 3rd, Bono CM, Gilbert TJ, et al. An evidence-based clinical guideline for the diagnosis and treatment of degenerative lumbar spondylolisthesis. *Spine J*. Jul 2009;9(7):609-14. doi:10.1016/j.spinee.2009.03.016
6. Austevoll IM, Hermansen E, Fagerland MW, et al. Decompression with or without Fusion in Degenerative Lumbar Spondylolisthesis. *New England Journal of Medicine*. 2021;385(6):526-538. doi:10.1056/NEJMoa2100990
7. Forsth P, Olafsson G, Carlsson T, et al. A Randomized, Controlled Trial of Fusion Surgery for Lumbar Spinal Stenosis. *N Engl J Med*. Apr 14 2016;374(15):1413-23. doi:10.1056/NEJMoa1513721
8. Inose H, Kato T, Yuasa M, et al. Comparison of Decompression, Decompression Plus Fusion, and Decompression Plus Stabilization for Degenerative Spondylolisthesis: A Prospective, Randomized Study. *Clin Spine Surg*. Aug 2018;31(7):E347-e352. doi:10.1097/bsd.0000000000000659
9. Frieden TR. Evidence for Health Decision Making — Beyond Randomized, Controlled Trials. *New England Journal of Medicine*. 2017;377(5):465-475. doi:10.1056/NEJMra1614394
10. Morse KW, Steinhaus M, Bovonratwet P, et al. Current treatment and decision-making factors leading to fusion vs decompression for one-level degenerative spondylolisthesis: survey results from members of the Lumbar Spine Research Society and Society of Minimally Invasive Spine Surgery. Spine J. Nov 2022;22(11):1778-1787. doi:10.1016/j.spinee.2022.07.095;
11. Agha RA, Altman DG, Rosin D. The SPIRIT 2013 statement--defining standard protocol items for trials. *Int J Surg*. Jan 2015;13:288-91. doi:10.1016/j.ijsu.2014.12.007
12. Gamble C, Krishan A, Stocken D, et al. Guidelines for the Content of Statistical Analysis Plans in Clinical Trials. *Jama*. Dec 19 2017;318(23):2337-2343. doi:10.1001/jama.2017.18556
13. Cuschieri S. The CONSORT statement. *Saudi J Anaesth*. Apr 2019;13(Suppl 1):S27-s30. doi:10.4103/sja.SJA_559_18
14. ICH, Harmonised, Tripartite, Guideline. Guideline for good clinical practice E6(R1). June 1996. http://wwwichorg/fileadmin/Public_Web_Site/ICH_Products/Guidelines/Efficacy/E6_R1/Step4/E6_R1__Guidelinepdf. 2014;
15. Dupuis PR, Yong-Hing K, Cassidy JD, Kirkaldy-Willis WH. Radiologic diagnosis of degenerative lumbar spinal instability. *Spine (Phila Pa 1976)*. Apr 1985;10(3):262-76.

16. Cho IY, Park SY, Park JH, Suh SW, Lee SH. MRI findings of lumbar spine instability in degenerative spondylolisthesis. *Journal of orthopaedic surgery (Hong Kong)*. May-Aug 2017;25(2):2309499017718907. doi:10.1177/2309499017718907

17. Berlemann UF, Jeszenszky DJ FAU, Buhler DW FAU, Harms J. - Facet joint remodeling in degenerative spondylolisthesis: an investigation of joint orientation and tropism. - *Eur Spine J1998;7(5):376-80*. 2005 2005:80. Not in File.

18. Masharawi Y, Kjaer P, Bendix T, et al. The reproducibility of quantitative measurements in lumbar magnetic resonance imaging of children from the general population. *Spine (Phila Pa 1976)*. Sep 01 2008;33(19):2094-100. doi:10.1097/BRS.0b013e31817f19f7

19. Schwab F, Lafage V, Patel A, Farcy JP. Sagittal plane considerations and the pelvis in the adult patient. *Spine (Phila Pa 1976)*. Aug 01 2009;34(17):1828-33. doi:10.1097/BRS.0b013e3181a13c08

20. Fairbank JC, Couper J, Davies JB, O'Brien JP. The Oswestry low back pain disability questionnaire. *Physiotherapy*. Aug 1980;66(8):271-3.

21. Baker DJ, Pynsent PB, J F. The Oswestry disability index revisited: its reliability, repeatability and validity, and a comparison with the St Thomas's disability index. *Roland MO, Jenner JR, eds New approaches to rehabilitation and education Manchester: Manchester University Press*. 1989:174-86.

22. Dworkin RH, Turk DC, Wyrwich KW, et al. Interpreting the clinical importance of treatment outcomes in chronic pain clinical trials: IMMPACT recommendations. *J Pain*. Feb 2008;9(2):105-21. doi:10.1016/j.jpain.2007.09.005

23. Ostelo RW, Deyo RA, Stratford P, et al. Interpreting change scores for pain and functional status in low back pain: towards international consensus regarding minimal important change. *Spine (Phila Pa 1976)*. Jan 1 2008;33(1):90-4. doi:10.1097/BRS.0b013e31815e3a10

24. Austevoll IM, Gjestad R, Grotle M, et al. Follow-up score, change score or percentage change score for determining clinical important outcome following surgery? An observational study from the Norwegian registry for Spine surgery evaluating patient reported outcome measures in lumbar spinal stenosis and lumbar degenerative spondylolisthesis. *BMC Musculoskelet Disord*. Jan 18 2019;20(1):31. doi:10.1186/s12891-018-2386-y

25. Fagerland MW, Lydersen S, Laake P. Recommended confidence intervals for two independent binomial proportions. *StatMethods MedRes*. 10/13/2011 2011;Not in File. doi:0962280211415469 [pii];10.1177/0962280211415469 [doi]

26. Rehal S, Morris TP, Fielding K, Carpenter JR, Phillips PP. Non-inferiority trials: are they inferior? A systematic review of reporting in major medical journals. *BMJ Open*. Oct 7 2016;6(10):e012594. doi:10.1136/bmjopen-2016-012594

27. Solberg TK, Sorlie A, Sjaavik K, Nygaard OP, Ingebrigtsen T. Would loss to follow-up bias the outcome evaluation of patients operated for degenerative disorders of the lumbar spine? *Acta Orthop*. 2/2011 2011;82(1):56-63. Not in File. doi:10.3109/17453674.2010.548024 [doi]

28. Yamaguchi Y, Ueno M, Maruo K, Gosho M. Multiple imputation for longitudinal data in the presence of heteroscedasticity between treatment groups. *Journal of Biopharmaceutical Statistics*. 2020/01/02 2020;30(1):178-196. doi:10.1080/10543406.2019.1632878

29. Floden L, Bell ML. Imputation strategies when a continuous outcome is to be dichotomized for responder analysis: a simulation study. *BMC Med Res Methodol*. Jul 23 2019;19(1):161. doi:10.1186/s12874-019-0793-x
