# Supplementary material for: Surgeon Recommendation and Outcomes of Decompression With vs Without Fusion in Patients With Degenerative Spondylolisthesis
Source: JAMA Netw Open. 2025 Jan 7;8(1):e2453466. doi: 10.1001/jamanetworkopen.2024.53466 (PMC11707628; doi:10.1001/jamanetworkopen.2024.53466)
Supplement: Supplement 2. — eAppendix. The NORDSTEN Collaboration eTable 1. Study Eligibility Criteria eTable 2. Surgical and Radiological Data (Modified Intention to Treat Population for the Total NORDSTEN-DS Trial) eTable 3. Associations of Baseline Characteristics and the Surgeon’s Preferred Treatment Analyzed With Multivariable Logistic Regression Models (N = 179) eTable 4. Proportion of Preference at Participating Departments eTable 5. Worsening According to GPE Scale and Subsequent Surgery From Index Surgery to 2-Year Follow-Up eReferences [file jamanetwopen-e2453466-s002.pdf]

# Supplementary Online Content

Seip A, Hellum C, Fagerland MW, et al. Surgeon recommendation and outcomes of decompression with vs without fusion in patients with degenerative spondylolisthesis. *JAMA Netw Open*. 2025;8(1):e2453466. doi:10.1001/jamanetworkopen.2024.53466

**eAppendix.** The NORDSTEN Collaboration

**eTable 1.** Study Eligibility Criteria

**eTable 2.** Surgical and Radiological Data (Modified Intention to Treat Population for the Total NORDSTEN-DS Trial)

**eTable 3.** Associations of Baseline Characteristics and the Surgeon's Preferred Treatment Analyzed With Multivariable Logistic Regression Models (N = 179)

**eTable 4.** Proportion of Preference at Participating Departments

**eTable 5.** Worsening According to GPE Scale and Subsequent Surgery From Index Surgery to 2-Year Follow-Up

**eReferences**

This supplementary material has been provided by the authors to give readers additional information about their work.

**eAppendix.** The NORDSTEN Collaboration

# 1. The NORDSTEN collaboration

The NORDSTEN collaboration is responsible for three ongoing studies: the NORDSTEN-DS trial, the NORDSTEN Lumbar Spinal Stenosis trial (NORDSTEN-SST), an RCT investigating three different techniques for decompression of lumbar spinal stenosis without spondylolisthesis; and the NORDSTEN Observational Cohort (NORDSTEN-OC) study, a prospective, longitudinal, observational study investigating the natural course of patients with spinal stenosis who did not undergo surgical treatment. So far, five Ph.D. candidates have been affiliated with the NORDSTEN studies, of which two have completed their degrees. The present study is based on the NORDSTEN-DS- database.

## 1.1. The organization

The Scientific Board is responsible for the academic component of the trials, drafting the study protocol and writing the scientific reports. The Administrative Executive Board is responsible for the administrative part of the trial, organizing the budget, applications for funding, and study logistics. The Working Committee consists of one surgeon (Principal Investigator (PI)) and one study coordinator from each participating hospital. Eleven orthopedic and five neurosurgical departments are participating representing every health region in Norway. The participating hospitals did 57% of the registered degenerative spine surgery in Norway in 2014. Private for-profit-hospitals did 19%, and the remaining 24% was done by public departments not participating in NORDSTEN.

It is founded by grants from from the Western Norway Regional Health Authority, the Møre and Romsdal Hospital Trust, and the Sophies Minde foundation. The R&D department at Møre and Romsdal Health Trust manages the trial finances. The Research and Communication Unit for Musculoskeletal Health (FORMI) at Oslo University Hospital (OUS) is responsible for coordinating the NORDSTEN study. The Working Committee, the Scientific Board, the Administrative Executive Board, and FORMI staff meet biannually for supervision, discussion, and adjustment of administrative routines. The surgeons and coordinators take part in teaching sessions to ensure compliance with protocols.

© 2025 Seip A et al. *JAMA Network Open.*

### 1.1.1.Scientific Board

**Leader:** Christian Hellum, MD, PhD

**Members:** Kjersti Storheim, PT, Prof; Kari Indrekvam, MD, Ass. Prof; Jens Ivar Brox, MD, Prof; Oliver Grundnes, MD, PhD; Inger Ljøstad, patient representative; Tore Solberg, MD, Prof; Ivar Magne Austevoll, MD, PhD; Erland Hermansen, MD, PhD; Frode Rekeland, MD; Jørn Aaen, MD; Clemens Weber, MD, PhD; Eric Franssen, MD; Hasan Banitalebi, MD,PhD; Masoud Anwar, MD, PhD

**International Collaborator**: Helena Brisby, MD, Prof

#### 1.1.2.Administrative Executive Board

**Leader:** Kari Indrekvam, MD, PhD

**Members:** Anniken Remseth, Bodil Røyset, Kjersti Storheim (FORMI), Christian Hellum, Erland Hermansen, Ivar M. Austevoll, and Frode Rekeland.

#### 1.1.3.Study Coordinating Center

FORMI, OUS is the study coordinating center, where data from all study centers is collected and administrated. FORMI coordinators communicate with the participating hospitals and ensure that all follow-up data is collected and stored according to research ethics laws.

**Leader**: Kjersti Storheim

**Research coordinators**; Ingrid Fjellheim Bånerud, Eira Kathleen Ebbs, Maren Hjelle Guddal, Marie Skovli Pettersen, Ørjan Nesse Vigdal.

#### 1.1.4.Data Safety/Monitory Board

Katarina Mølsæter is responsible for monitoring the study.

© 2025 Seip A et al. *JAMA Network Open.*

# 2.Trial investigators

**Participating hospitals with numbers of patients included in** NORDSTEN-DS**:**

| **Hospital** | **Number of patients** |
|---|---|
| Akershus University Hospital, Orthopedic department | 12 |
| Haukeland University Hospital, Kysthospitalet i Hagevik, Orthopedic department | 78 |
| Haukeland University Hospital, Neurosurgical department | 9 |
| Haukeland University Hospital, Orthopedic department | 13 |
| Innlandet Hospital Trust, Division Elverum, Orthopedic department | 2 |
| Innlandet Hospital Trust, Division Gjøvik, Orthopedic department | 12 |
| Innlandet Hospital Trust, Division Lillehammer, Orthopedic department | 2 |
| Møre and Romsdal Hospital Trust, Ålesund Hospital, Orthopedic department | 24 |
| Oslo University Hospital, Ullevål, Orthopedic Division | 25 |
| Stavanger University Hospital, Orthopedic department and Neurosurgical department | 42 |
| St. Olav’s Hospital, Neurosurgical department | 4 |
| Sørlandet Hospital Trust, Arendal Hospital, Orthopedic department | 7 |
| Sørlandet Hospital Trust, Kristiansand Hospital, Orthopedic department | 1 |
| Telemark Hospital Trust, Skien Hospital, Orthopedic department | 12 |
| University Hospital of Northern Norway, Neurosurgical department | 13 |
| Vestre Viken Hospital Trust, Bærum Hospital, Orthopedic department | 11 |
| **Total** | **267** |

© 2025 Seip A et al. *JAMA Network Open.*

## 2.1.Participating surgeons and coordinators

**Akershus University Hospital, Orthopedic department**

Oliver Grundnes, Therese Gundersen, Trine Myrvold, Ole Kristian Alhaug, Sverre Mjønes, Abdullah Cetinkaia, Filip Dolatowski, Christina Handeland

**Haukeland University Hospital, Kysthospitalet i Hagevik, Orthopedic department**

Arild Ulvestad, Turid Rognsvåg, Janne Haugland, Eva Saltskår Jentoft, Truls Rokne Hanestad, Eric Kgomotso, Magnus Valland, Ørjan Stensletten, Ivar Magne Austevoll, Erland Hermansen, Mohammed Ahmed, Andreas Seip, Frode Rekeland, Rune Birketvedt

**Haukeland University Hospital, Neurosurgical department**
Samer Habiba, Kristine Helland, Linda Sæterdal

**Haukeland University Hospital, Orthopedic department**
Maria Rieber-Mohn, Andreas Seip, Truls Hanestad, Sylvi Ann Sagstad, Per Torgeir Nilsen, Ivar Magne Austevoll

**Innlandet Hospital Trust, Division Elverum, Orthopedic department**
Arnfinn Pedersen, Hege Westgård, Anders Tønsager

**Innlandet Hospital Trust, Division Gjøvik, Orthopedic department**
Håvard Furunes, Espen Mørk, Inger Opheim, Hege Bergum Nilsen, Øyvind Sletten, Aina Strøm, Eirik Østvold Melandsø, Maria Van My Nguyen Giebers

**Innlandet Hospital Trust, Division Lillehammer, Orthopedic department**
Vinjar Hansen, Greger Lønne, Roar Rønning, Gisle Szacinski

**Møre and Romsdal Hospital Trust, Ålesund Hospital, Orthopedic department**
Erland Hermansen, Jørn Aaen, Vidar Punsvik, Anita Karin Vassbakk, Alf Hellevik

**Oslo University Hospital, Ullevål, Orthopedic Division and Division of Clinical Neuroscience**

© 2025 Seip A et al. *JAMA Network Open.*

Christian Hellum, Espen Sjåberg, Ellen Aksnes, Roger Trana, Ole Rasmus Robak, Morten Karlsen, Jon Magne Hoseth, Ali Al-Ashtari, Geir Jomaas, Marte Magnusson, Maren Hjelle Guddal, Ingrid Fjellheim Bånerud, Eira Ebbs, Marie Skovli Pettersen, Kjersti Storheim, Ørjan Nesse Vigdal, Mads Rolfsen, Vinjar Myklevoll, Trine Strøm, Hege C. Thrygg

**Stavanger University Hospital, Orthopedic department and Neurosurgical department**
Eric Franssen, Knut Harboe, Elisabeth Rettedal, Kirstine Eikenes, Gabriel Lende, Clemens Weber, Hilde Olsen

**St. Olav's Hospital, Neurosurgical department**
Clemens Weber, Øyvind Sletten, Tomm Müller, Camilla Brattbakk, Hege Andresen, Øystein Nygaard

**Sørlandet Hospital Trust, Arendal Hospital, Orthopedic department**
Pål Amesen, Yngve Sporstøl, Ted P. Lundgren, Anne-Charlotte Fosse Haug, Elisabeth Lilleholdt Muller

**Sørlandet Hospital Trust, Kristiansand Hospital, Orthopedic department**
Odd Arild Ågedal, Kjartan Krogedal

**Telemark Hospital Trust, Skien Hospital, Orthopedic department**
Knut Jørgen Haug, Yngve Sporstøl, Ingrid Edbo

**University Hospital of Northern Norway, Neurosurgical department**
Tore Solberg, Rune Hennig, Andreas Sørlie, Jørgen Isaksen, Jens Munch-Ellingsen, Kristine Evanger, Caroline W. Thórisdóttir, Vibeke Grønaas, Marilyn Botn, Silje M. Nilssen, Lasse Andreassen, Sunniva Andreassen, Sara Kristin Sørend

**Vestre Viken Hospital Trust, Bærum Hospital, Orthopedic department**
Vidar Opland, Merete Finjarn, Ellen Langslet, Wender Figved, Lars Forberg, Morten Karlsen, Erik Øygarden, Lauritz Dahl

© 2025 Seip A et al. *JAMA Network Open.*

# 3.Patient representation and involvement

The patient representative in the NORDSTEN study is Inger Ljøstad. She is affiliated with the Norwegian Back and Spine Association (Ryggforeningen), a membership organization which mainly comprises people suffering from back pain.

The patient representative is a member of the study's Steering Committee and attends meetings and sessions in both the Steering Committee and Working Group and takes part in discussions to make sure the patients' perspectives and involvement are implemented in applications and in the NORDSTEN trials. She also provides contact between the researchers and the Norwegian Back and Spine Association and contributes to the newsletters sent to study participants twice a year.

In the present study our patient representative has been involved in the drafting and revision of this manuscript.

## 4.Implementation of a modified model of Good Clinical Practice in the NORDSTEN-DS trial

To ensure ethical research for participants, enhance the quality of obtained data, and to initiate good research structure a modified model of Good Clinical Practice Guidelines was developed for the Nordsten-DS trial, inspired by The International Conference on Harmonization of Good Clinical Practice Guidelines. Details are to be found elsewhere.[1]

© 2025 Seip A et al. *JAMA Network Open.*

# 5.Supplement to the Methods

## 5.1.Outcome measurements

Outcome measurements were collected by questionnaires through mail, and data regarding radiology and reoperations were collected at follow-up visits by study coordinators.

### 5.1.1.Patient-reported outcome measurements

#### *Oswestry Disability Index (ODI) V.2.0*

ODI comprises 10 items with six alternatives presented in increasing order of disability, connected to pain and pain-related disability in daily activities.[2] Zero represents no disability and ten represents the greatest impairment. For each item, the ODI score is the sum of the responses divided by the number of items responded to multiplied by 10. Hence, total ODI scores range from 0 to 100, where 100 represents the greatest impairment.

The present version has been translated into Norwegian, and its reliability and validity have been found to be acceptable for the assessment of functional disability in the Norwegian population.[3]

#### *Zürich Claudication Questionnaire (ZCQ)*

ZCQ is a spinal stenosis specific score with three domains: symptom severity (7 items ranging from 1 to 5), physical function (5 items ranging from 1 to 4), and patient satisfaction (6 items ranging from 1 to 4). A score of one represents the lowest symptom severity, least physical impairment, and highest patient satisfaction.[4] The ZCQ has been translated into Norwegian.[5] A methodology study found the reliability of the scale to be ‘very good’, the validity ‘acceptable’, and the responsiveness to be ‘good’.[5]

#### *Numerical Rating Scales for leg and back pain*

NRS is a measurement of pain experienced in the last week. Patients rate their pain during the last week ranging from 0 (no pain) to 10 (the maximum imaginable pain), i.e. an 11-point

© 2025 Seip A et al. *JAMA Network Open.*

Likert scale,.[6] The measurement has acceptable reliability and validity,[7] is easy to understand, and has shown high test-retest reliability [8].

## *EuroQol 5 dimensional descriptive system (EQ-5D-3L)*

EQ-5D is a generic questionnaire comprising mobility, self-care, usual activity, pain/discomfort, and anxiety/depression [9]. The five questions has a three-point descriptive scale where 1 represents the best possible healthEach possible combination of responses ($3^5$ = 243) represents a score between -0.59 and 1.0, with higher scores indicating better quality of life. . The UK value set is used in the scoring algorithm. Its validity, reliability, and responsiveness have been evaluated and found to be acceptable for Norwegian patients operated for lumbar degenerative disorders.[10]

## *Radiological exams.*

1. Magnetic Resonance Imaging (MRI) of the lumbar spine less than 6 months before surgery.
2. X-ray less than 6 months before surgery.

*MRI*

In accordance with the radiological protocol, we performed sagittal T1- and axial and sagittal T2- weighted images with repetition time (TR)/ echo time (TE) 400 - 826 ms/ 8 - 14 ms for T1-weighted images and 1500 - 6548 ms/ 82 -126 ms for T2-weighted images, slice thickness: 3 - 5 mm, FOV:160 - 350 mm.

*X-rays*

In accordance with the radiological protocol, exams were performed in a standing position in frontal and lateral view and with maximum flexion and extension view.

*Evaluation of exams*

Two consultant orthopedic spine surgeons and two consultant radiologists evaluated the exams. This was done independently, blinded to each other’s evaluations and the clinical symptoms. The preoperative images were evaluated and reported on first, followed by the postoperative images. All observers used PACS (Picture Archiving and Communication System IDS7, Sectra, Sweden). Each exam was at least evaluated by two observers (one surgeon and one radiologist). The mean observer value defined the values of continuous

© 2025 Seip A et al. *JAMA Network Open.*

variables whereas a majority decision defined values of categorized variables (at least three observers were required in cases with disagreement between two observers)

**Assignment to agreement and disagreement group:**

As part of the inclusion papers for the NORDSTEN-DS trial, the surgeon had to answer: *Hvilken behandling mener du ville vært best for denne pasienten, dersom han/hun ikke var del av studien?*

Our translation to English: *Which treatment do you consider best for this patient, if he/she had not been participating in this trial?*

## 5.2. Statistical analysis

### 5.2.1. Management of missing data

Under the assumption of missing at random (MAR), missing values necessary for calculating responder rates at two-year follow-up were estimated by multiple imputation (MI). The MAR assumption for patients not replying to PROM questionnaires was supported by a previous study from the Norwegian Registry for Spine Surgery.[12] Further, due to a comprehensive set of available predictors for the imputation model, we considered the MI method to be robust against biased estimates. In the imputation model, using linear regression for continuous variables and logistic regression for dichotomous variables, we included the following 27 explanatory variables: age, gender, smoking, former spinal surgery, and baseline and follow-up scores (3 months, 1 year and 2 years) for ODI, ZCQ symptom severity, ZCQ physical function, ZCQ patient satisfaction, NRS leg pain, and NRS back pain. We tried to add a greater number of explanatory variables, however, numerical problems made estimation of the resulting imputation models impossible. The imputations were stratified by treatment (i.e., separately by treatment group).[13] As recommended, the imputations were performed before dichotomizing the responder variables.[14] Imputed 2-year follow-up scores for the ODI were estimated based on 50 generated data sets.

Primary outcome:

© 2025 Seip A et al. *JAMA Network Open.*

For the primary outcome we followed the same statistical plan as in the original study, with tree sensitivity analyses: Per-protocol population, complete cases, and last with missing data at 2-year follow up replaced with data at 1 year.

## **eTable 1.** Study Eligibility Criteria

| **Inclusion criteria** | **Exclusion criteria** |
|---|---|
| • Over 18 years of age and able to give informed consent.<br>• Understand Norwegian language, spoken and written.<br>• Spondylolisthesis, with a slip >=3 mm, verified on standing lateral x-rays.<br>• Spinal stenosis at the level of spondylolisthesis, shown on MRI, CT scan, and/or myelography.<br>• Clinical symptoms of spinal stenosis, defined as neurogenic claudication or radiating pain into the lower limbs, not responding to at least 3 months of non-operative treatment.<br>• Able to respond to the questionnaires. | • Not willing to give written consent.<br>• Participating in another clinical trial that may interfere with this trial.<br>• ASA- grade >3 [a]<br>• Older than 80 years.<br>• Not able to fully comply with the protocol, including treatment, follow-up, or study procedures (psychosocially, mentally, and physically).<br>• Cauda equina syndrome (bowel or bladder dysfunction) or complete motor deficit.<br>• A slip >=3 mm in more than one level.<br>• Isthmic defect in pars interarticularis.<br>• Fracture or former fusion of the thoracolumbar region.<br>• Previous surgery in the level of spondylolisthesis.<br>• A lumbosacral scoliosis of more than 20 degrees verified on AP view.<br>• Distinct symptoms in one or both legs due to other diseases, e.g. polynevropathy, vascular claudication, or osteoarthritis.<br>• ODI score <25 [b]<br>• Radicular pain due to a foraminal stenosis at the slipped level with deformation of the nerve root because of a bony narrowing in the vertical direction, verified by MRI. [c] |

[a] An American Society of Anesthesiologists (ASA) score of 1 indicates the presence of no disease, 2 the presence of mild systemic disease, and 3 the presence of severe systemic disease that is not life-threatening.
[b] From February 12, 2014 (start of inclusion) to August 29, 2015, a score below 25 on the Oswestry Disability Index was an exclusion criterion (see Amendment in the published study protocol).[15][c] Grade 3 according to Lee Classification.[16]

© 2025 Seip A et al. *JAMA Network Open.*

**eTable 2.** Surgical and Radiological Data (Modified Intention to Treat Population for the Total NORDSTEN-DS Trial)[a]

| | Decompression alone | Decompression with instrumented fusion |
|---|---|---|
| **Surgical data** | | |
| Level of spondylolisthesis – no./total no (%) | | |
| L2/L3 | 1/133 (1) | 2/129 (2) |
| L3/L4 | 16/133 (12) | 19/129 (15) |
| L4/L5 | 112/133 (84) | 107/129 (83) |
| L5/S1 | 4/133 (3) | 1/129 (1) |
| Number of levels decompressed – no./total no (%) | | |
| One level | 111/133 (83) | 105/128 (82) |
| Two levels | 21/133 (16) | 21/128 (16) |
| Three levels | 1/133 (1) | 2/128 (1.6) |
| Operated level [b] – no./total no (%) | | |
| L2/L3 | 2/132 (2) | 3/124 (2) |
| L3/L4 | 28/132 (21) | 33/124 (27) |
| L4/L5 | 115/132 (87) | 107/124 (86) |
| L5/S1 | 7/132 (5) | 3/124 (2) |
| Decompression method – no./total no (%) | | |
| Midline structures preserved | 130/133(98) | 58/128 (45) |
| Midline structures not preserved | 3/133 (2) | 70/128 (55) |
| Use of microscope or lenses – no./total no (%) | 127/133 (95) | 107/124 (86) |
| Instrumented fusion – no./total no (%) | | |
| Including interbody fusion | | 47/124 (38) |
| Without interbody fusion | | 77/124 (62) |
| Graft – No./total no (%) | | |
| Autograft | | 111/111 (100) |
| Allograft | | 1/111 (1) |
| Xenograft | | 13/111 (12) |
| Use of wound drain – no./total no (%) | 61/124 (49) | 76/121 (63) |

© 2025 Seip A et al. *JAMA Network Open.*

| | Decompression alone | Decompression with instrumented fusion |
|---|---|---|
| **Radiological parameters** | | |
| Degree of spondylolisthesis [c] – mm | 7.6 (± 3.2) | 7.2 (± 2.8) |
| Facet joint fluid [d] – mm | 1.1 (± 1.0) | 1.2 (± 1.0) |
| Facet joint fluid >2mm – no./total no (%) | 24/125 (19) | 25/125 (20) |
| Modic changes [e] – no./total no (%) | 23/127 (18) | 15/124 (12) |
| Disc degeneration [f] – no./total no (%) | 12/129 (9) | 8/125 (6) |
| Foraminal stenosis [g] – no./total no (%) | 12/111 (11) | 11/113 (10) |
| Segmental instability [h] – no./total no (%) | | |
| ≥3 mm forward translation | 26/121 (22) | 19/112 (17) |
| ≥ 10 degrees loss of lordosis | 9/121 (7) | 8/119 (7) |
| Orientation of the facet joint [i] – degrees | 56 ± 9 | 57 ± 9 |
| Disc height in the level of olisthesis [j] – mm | 7.6 ±2.0 | 8.0 ± 2.1 |
| Lumbal lordosis [k] – degrees | 54 ± 11 | 54 ± 11 |

[a] A modified intention-to-treat cohort consisted of all participants who received the trial treatment in accordance with the randomization and had available data after randomization. Plus-minus values are means ±SD.

[b] Including levels decompressed without spondylolisthesis.

[c] Refers to the degree of spondylolisthesis in standing x-rays in millimeters [17]

[d] Refers to the fluid gap between upper end lower facet at the level of spondylolisthesis assessed on the MRI axial plane.[18]

[e] Refers to Modic type I, type II, and mixed type I and II, verified on MRI.[19]

[f] Refers to grade 5 according to Pfirmann classification (ranging from 1 to 5, where higher grade indicates more extensive degeneration).[20]

[g] Refers to foraminal stenosis grades 2 and 3 according to Lee classification (ranging from 0 to 3, where higher grade indicates more extensive stenosis). [16]

[h] Refers to commonly used criteria for instability assessed on standing X-ray (extension minus flexion).[17,21]

[i] Refers to the angle (mean of right and left joint assessed by MRI, axial plane) at the level of spondylolisthesis.[22]

[j] Refers to the middle disc height (distance between mid-inferior and mid-superior disc borders assessed on a mid-sagittal MRI plane). [23]

[k] Refers to the angle between upper endplate S1 and lower endplate L1 on standing x-ray. [24]

© 2025 Seip A et al. *JAMA Network Open.*

**eTable 3.** Associations of Baseline Characteristics and the Surgeon's Preferred Treatment Analyzed With Multivariable Logistic Regression Models (N = 179)[a]

| Covariate | Odds ratio (95% CI) | p-value |
|---|---|---|
| Age – years, continue | 0.99 (0.94 – 1.03) | 0.58 |
| Sex – **male**/female | 1.11 (0.53 – 2.33) | 0.79 |
| Smoking - **no**/yes | 0.62 (0.24 – 1.55) | 0.30 |
| Body-mass index (continue) | 1.03 (0.95 -1.12) | 0.50 |
| ASA score (**1**,2,3) [b] | 0.87 (0.42 – 1.78) | 0.69 |
| Predominant back pain – continue [c] | 1.16 (0.95 – 1.55) | 0.15 |
| Degree of spondylolisthesis - mm | 1.28 (1.13 – 1.45) | <0.001 |
| Dynamic segmental instability [d] | | |
| Forward translation – mm [e] | 0.94 (0.77 – 1.14) | 0.53 |
| Segmental loss of lordosis - degrees | 1.13 (1.02 – 1.25) | 0.022 |
| Facet joint fluid–mm [f] | 0.79 (0.56 - 1.11) | 0.17 |
| Orientation of the facet joint – degrees | 1.01 (0.97 – 1,06) | 0.55 |
| Lumbar lordosis – degrees [g] | 1.01 (0.98 – 1.05) | 0.45 |
| Disc heigth – mm [h] | 0.93 (0.79 – 1.11) | 0.44 |

[a] Associations of baseline characteristics and surgeon preference for supporting decompression with instrumental fusion in 179 patients with intact data on all variables. The model includes previous suggested and clinically plausible variables which potentially were associated with the dependent variable.
ASA (ref = no comorbidity), Sex (ref = females), Smoking (ref = no).
[b] ASA = American Society of Anesthesiologists Classification
[c] Predominant backpain is NRS backpain minus NRS leg pain, as a continuous variable. NRS is Numerical Rating Scale, which range from 0 to 10, where lower values indicate less pain
[d] Dynamic segmental instability assessed on standing standard radiographs when bending forward from extended position.
[e] Forward translation refers to the forward translation of the upper vertebra relative to the vertebra below assessed on standing standard radiographs in millimetre.
[f] Facet joint fluid refers to the assessed gap between the upper end lower facet at the level of spondylolisthesis assessed on the MRI axial plane.
[g] Lumbar lordosis refers to the angle between upper endplate S1 and lower endplate L1 on standing standard radiographs.
[h] Disc height in the level of olisthesis refers to the distance between mid-inferior and mid-superior disc borders assessed on a mid-sagittal MRI plane.

© 2025 Seip A et al. *JAMA Network Open.*

**eTable 4.** Proportion of Preference at Participating Departments

| Department | Included in NORDSTEN-DS | Preferred decompression alone | Preferred dekompression + fusion | Preferred to decompression | Response rate |
|---|---|---|---|---|---|
| 1-4[a] | 8 | 4 | 2 | 67 % | 75 % |
| 5 | 7 | 3 | 4 | 43 % | 100 % |
| 6 | 9 | 4 | 2 | 67 % | 67 % |
| 7 | 11 | 4 | 5 | 44 % | 82 % |
| 8 | 12 | 10 | 0 | 100 % | 83 % |
| 9 | 12 | 6 | 4 | 60 % | 83 % |
| 10 | 12 | 6 | 4 | 60 % | 83 % |
| 11 | 13 | 9 | 0 | 100 % | 69 % |
| 12 | 13 | 3 | 6 | 33 % | 69 % |
| 13 | 24 | 7 | 15 | 32 % | 92 % |
| 14 | 25 | 7 | 12 | 37 % | 76 % |
| 15 | 42 | 19 | 17 | 53 % | 86 % |
| 16 | 78 | 30 | 39 | 43 % | 88 % |
| Total | 267 | 112 | 110 | 50 % | 83 % |

[a]Four departments with 1-4 included patients.

© 2025 Seip A et al. *JAMA Network Open.*

## **eTable 5.** Worsening According to GPE Scale and Subsequent Surgery From Index Surgery to 2-Year Follow-Up[a]

[a] Modified intention-to-treat set

| | Agreement group[b] | Disagreement group |
|---|---|---|
| **Worsening symptoms according to GPE scale** | 7/105 (7) | 7/100 (7) |
| **Subsequent lumbar surgery within 2 years after surgery** | 7/114 (6) | 15/106 (14) |
| **Main reason for reoperation** | | |
| Subfascial infection | 1 | 4 |
| Hematoma | 1 | 1 |
| Surgery on wrong side/level | 0 | 1 |
| Leg or back pain due to re-stenosis at index level | 2 | 6 |
| Leg or back pain due to stenosis at a new lumbar level | 1 | 0 |
| Incomplete decompression (index level) | 0 | 1 |
| Pedicle screw misplacement | 0 | 1 |
| Back pain due to disc degeneration, spondylolisthesis | 1 | 0 |
| Pain due to implants/pseudoarthrosis/screw loosening | 1 | 1 |
| **Main type of surgery** | | |
| Wound revision without fusion surgery | 1 | 4 |
| Decompression surgery without fusion | 1 | 4 |
| Fusion surgery and decompression | 0 | 3 |
| Fusion surgery without decompression | 4 | 4 |
| Removal of implants due to back pain | 1 | 0 |

[b] Values are numbers (percentage)

© 2025 Seip A et al. *JAMA Network Open.*

## eReferences


1. Austevoll IM, Hermansen E, Fagerland MW, et al. Decompression with or without Fusion in Degenerative Lumbar Spondylolisthesis. New England Journal of Medicine 2021;385(6):526-538. DOI: 10.1056/NEJMoa2100990.
2. Fairbank JC, Couper J, Davies JB, O'Brien JP. The Oswestry low back pain disability questionnaire. Physiotherapy 1980;66(8):271-3. (In eng).
3. Grotle M, Brox JI, Vollestad NK. Cross-cultural adaptation of the Norwegian versions of the Roland-Morris Disability Questionnaire and the Oswestry Disability Index. JRehabilMed 2003;35(5):241-247. (http://www.ncbi.nlm.nih.gov/pubmed/14582557).
4. Tuli SK, Yerby SA, Katz JN. Methodological approaches to developing criteria for improvement in lumbar spinal stenosis surgery. Spine (Phila Pa 1976) 2006;31(11):1276-80. (In eng). DOI: 10.1097/01.brs.0000217615.20018.6c.
5. Thornes E, Grotle M. Cross-cultural adaptation of the Norwegian version of the spinal stenosis measure. EurSpine J 2008;17(3):456-462. DOI: 10.1007/s00586-007-0576-7 [doi].
6. Hjermstad MJ, Fayers PM, Haugen DF, et al. Studies comparing Numerical Rating Scales, Verbal Rating Scales, and Visual Analogue Scales for assessment of pain intensity in adults: a systematic literature review. J Pain Symptom Manage 2011;41(6):1073-93. (In eng). DOI: 10.1016/j.jpainsymman.2010.08.016.
7. Froud R, Fawkes C, Foss J, Underwood M, Carnes D. Responsiveness, Reliability, and Minimally Important and Minimal Detectable Changes of 3 Electronic Patient-Reported Outcome Measures for Low Back Pain: Validation Study. J Med Internet Res 2018;20(10):e272. (In eng). DOI: 10.2196/jmir.9828.
8. Gallasch CH, Alexandre NM. The measurement of musculoskeletal pain intensity: a comparison of four methods. Rev Gaucha Enferm 2007;28(2):260-5. (In eng).
9. EuroQol--a new facility for the measurement of health-related quality of life. Health Policy 1990;16(3):199-208. (In eng).
10. Solberg TK, Olsen JA, Ingebrigtsen T, Hofoss D, Nygaard OP. Health-related quality of life assessment by the EuroQol-5D can provide cost-utility data in the field of low-back surgery. Eur Spine J 2005;14(10):1000-7. (In eng). DOI: 10.1007/s00586-005-0898-2.
11. Lonne G, Johnsen LG, Aas E, Lydersen S, Andresen H, Ronning R, Nygaard OP. Comparing cost-effectiveness of X-Stop with minimally invasive decompression in lumbar spinal stenosis: a randomized controlled trial. Spine (Phila Pa 1976) 2015;40(8):514-20. (In eng). DOI: 10.1097/brs.0000000000000798.
12. Solberg TK, Sorlie A, Sjaavik K, Nygaard OP, Ingebrigtsen T. Would loss to follow-up bias the outcome evaluation of patients operated for degenerative disorders of the lumbar spine? Acta Orthop 2011;82(1):56-63. DOI: 10.3109/17453674.2010.548024 [doi].
13. Yamaguchi Y, Ueno M, Maruo K, Gosho M. Multiple imputation for longitudinal data in the presence of heteroscedasticity between treatment


© 2025 Seip A et al. *JAMA Network Open.*

groups. J Biopharm Stat 2020;30(1):178-196. (In eng). DOI: 10.1080/10543406.2019.1632878.
14. Floden L, Bell ML. Imputation strategies when a continuous outcome is to be dichotomized for responder analysis: a simulation study. BMC Med Res Methodol 2019;19(1):161. (In eng). DOI: 10.1186/s12874-019-0793-x.
15. Austevoll IM, Hermansen E, Fagerland M, et al. Decompression alone versus decompression with instrumental fusion the NORDSTEN degenerative spondylolisthesis trial (NORDSTEN-DS); study protocol for a randomized controlled trial. BMC Musculoskelet Disord 2019;20(1):7. (In eng). DOI: 10.1186/s12891-018-2384-0.
16. Lee S, Lee JW, Yeom JS, Kim KJ, Kim HJ, Chung SK, Kang HS. A practical MRI grading system for lumbar foraminal stenosis. AJR AmJRoentgenol 2010;194(4):1095-1098. DOI: 194/4/1095 [pii];10.2214/AJR.09.2772 [doi].
17. Dupuis PR, Yong-Hing K, Cassidy JD, Kirkaldy-Willis WH. Radiologic diagnosis of degenerative lumbar spinal instability. Spine (Phila Pa 1976) 1985;10(3):262-76. (In eng).
18. Cho IY, Park SY, Park JH, Suh SW, Lee SH. MRI findings of lumbar spine instability in degenerative spondylolisthesis. Journal of orthopaedic surgery (Hong Kong) 2017;25(2):2309499017718907. (In eng). DOI: 10.1177/2309499017718907.
19. Modic MT, Steinberg PM, Ross JS, Masaryk TJ, Carter JR. Degenerative disk disease: assessment of changes in vertebral body marrow with MR imaging. Radiology 1988;166(1 Pt 1):193-9. (In eng). DOI: 10.1148/radiology.166.1.3336678.
20. Pfirrmann CW, Metzdorf A, Zanetti M, Hodler J, Boos N. Magnetic resonance classification of lumbar intervertebral disc degeneration. Spine (Phila Pa 1976) 2001;26(17):1873-1878. (http://www.ncbi.nlm.nih.gov/pubmed/11568697).
21. Weinstein JN, Lurie JD, Tosteson TD, et al. Surgical versus nonsurgical treatment for lumbar degenerative spondylolisthesis. N Engl J Med 2007;356(22):2257-70. (In eng). DOI: 10.1056/NEJMoa070302.
22. Berlemann UF, Jeszenszky DJ FAU, Buhler DW FAU, Harms J. - Facet joint remodeling in degenerative spondylolisthesis: an investigation of joint orientation and tropism. - Eur Spine J1998;7(5):376-80 2005:80. (http://download.springer.com/static/pdf/453/art%253A10.1007%252Fs005860050093.pdf?auth66=1364216598_f44772bbd1874f48625153a519562548&ext=.pdf).
23. Masharawi Y, Kjaer P, Bendix T, et al. The reproducibility of quantitative measurements in lumbar magnetic resonance imaging of children from the general population. Spine (Phila Pa 1976) 2008;33(19):2094-100. (In eng). DOI: 10.1097/BRS.0b013e31817f19f7.
24. Schwab F, Lafage V, Patel A, Farcy JP. Sagittal plane considerations and the pelvis in the adult patient. Spine (Phila Pa 1976) 2009;34(17):1828-33. (In eng). DOI: 10.1097/BRS.0b013e3181a13c08.

© 2025 Seip A et al. *JAMA Network Open.*
