# Supplementary material for: Surgeon Recommendation and Outcomes of Decompression With vs Without Fusion in Patients With Degenerative Spondylolisthesis
Source: JAMA Netw Open. 2025 Jan 7;8(1):e2453466. doi: 10.1001/jamanetworkopen.2024.53466 (PMC11707628; doi:10.1001/jamanetworkopen.2024.53466)
Supplement: Supplement 3. — Data Sharing Statement [file jamanetwopen-e2453466-s003.pdf]

Data Sharing Statement

Seip. Surgeon Recommendation and Outcomes of Decompression With vs Without Fusion in Patients With Degenerative Spondylolisthesis. *JAMA Netw Open*. Published January 07, 2025. doi:10.1001/jamanetworkopen.2024.53466

Data
Data available: Yes
Data types: Deidentified participant data
How to access data: By request to imau@helse-bergen.no
When available: With publication

Supporting Documents
Document types: None

Additional Information
Who can access the data: Data will be available to medical researchers by request.
Types of analyses: Data will be provided following review and approval of a research proposal and Statistical Analysis Plan (SAP)in accordance with local registration and ethical approval.
Mechanisms of data availability: Proposals requesting data access will need approval of the scientific board before any data can be released.
Any additional restrictions: The data will be accessible for 12 months, with possible extensions considered.
